# Associations between systemic inflammation and cognitive trajectories post-stroke

**DOI:** 10.1038/s41598-025-27119-1

**Published:** 2025-11-28

**Authors:** Heidi Vihovde Sandvig, Ingvild Saltvedt, Trine Holt Edwin, Stina Aam, Katinka Nordheim Alme, Rannveig Sakshaug Eldholm, Stian Lydersen, Tom Eirik Mollnes, Ragnhild Munthe-Kaas, Per Magne Ueland, Arve Ulvik, Torgeir Wethal, Anne-Brita Knapskog

**Affiliations:** 1 Department of Medicine, Kristiansund Hospital, Sjukehuset Nordmøre og Romsdal, Møre og Romsdal Hospital Trust, Kristiansund, Norway; 2 Department of Neuromedicine and Movement Science, Faculty of Medicine and Health Sciences, NTNU – Norwegian University of Science and Technology, Trondheim, Norway; 3 Department of Geriatric Medicine, Clinic of Medicine, St. Olavs Hospital, Trondheim University Hospital, Trondheim, Norway; 4 Department of Geriatric Medicine, Oslo University Hospital, Ullevaal, Oslo Norway; 5 Department of Internal Medicine, Haraldsplass Deaconess Hospital, Bergen, Norway; 6 Department of Mental Health, Faculty of Medicine and Health Sciences, NTNU – Norwegian University of Science and Technology, Trondheim, Norway; 7 Department of Immunology, Oslo University Hospital and University of Oslo, Oslo, Norway; 8 Research Laboratory, Nordland Hospital, Bodø, Norway; 9 Centre of Molecular Inflammation Research, NTNU – Norwegian University of Science and Technology, Trondheim, Norway; 10 Department of Medicine, Kongsberg Hospital, Vestre Viken Hospital Trust, Drammen, Norway; 11 Department of Medicine, Baerum Hospital, Vestre Viken Hospital Trust, Drammen, Norway; 12 Bevital A/S, Laboratoriebygget, Bergen, 5021 Norway; 13 Department of Stroke, Clinic of Medicine, St. Olavs Hospital, Trondheim University Hospital, Trondheim, Norway

**Keywords:** Cognitive trajectories, Ischemic stroke, Systemic inflammation, Frailty, The terminal C5b-9 complement complex, Tumour necrosis factor, Dementia, Stroke

## Abstract

Our objective was to explore whether plasma inflammatory biomarkers and related metabolites in acute phase and 3 months after stroke were associated with different cognitive trajectories and with changes in cognition post-stroke. The Norwegian Cognitive Impairment After Stroke (Nor-COAST) study was a prospective, multicentre cohort study of patients with acute stroke, followed up at 3, 18, and 36 months post-stroke. First, we modelled cognitive trajectory groups based on Montreal Cognitive Assessment (MoCA) scores and used multinominal logistic regression to study the associations between systemic inflammatory biomarkers/metabolites and group membership. Second, using mixed linear regression, we investigated whether the same biomarkers/metabolites were associated with changes in MoCA scores over time, stratified by pre-stroke cognitive status. The 466 participants had mean (SD) age 72 (12) years, 59% were males, and mean (SD) NIHSS score at admittance was 4 (4.8). Higher acute-phase values of the terminal complement complex, interleukin 6, macrophage inflammatory protein 1α, neopterin, quinolinic acid, and PA ratio = 4-pyridoxic acid / (pyridoxal + pyridoxal 5’-phosphate) and higher 3-month values of neopterin were associated with increased risk of being in the group characterized by low and declining MoCA score compared to the group of best MoCA score (*p* < 0.01). Higher acute-phase values of tumour necrosis factor and interleukin 8, were associated with progressive decline in the MoCA score (*p* < 0.01). Premorbid factors, and in particular pre-stroke frailty, had more impact on the models than stroke-related factors, and partly confounded several of these associations. Higher degrees of systemic inflammation in the acute phase were associated with worse cognitive trajectories and may reflect the response to the acute stroke, stroke-related complications and/or premorbid conditions.

Trial registration: ClinicalTrials.gov: NCT02650531. Retrospectively registered January 8, 2016. First participant included May 18, 2015.

**Supplementary Information:**

The online version contains supplementary material available at 10.1038/s41598-025-27119-1.

## Introduction

Post-stroke cognitive impairment (PSCI) can follow the acute neuronal death caused by the stroke, but stroke is also associated with an accelerated cognitive decline over time^1–3^. A prior publication from our multicentre, prospective cohort of stroke survivors, the Norwegian Cognitive Impairment After Stroke (Nor-COAST) study, showed that global cognitive functions from 3 to 18 months post-stroke were stable^4^, while another Norwegian study of patients with minor strokes found an improvement of cognitive function between 3 and 12 months post-stroke^5^. Other studies indicate that there is a turning point in the cognitive slope leading to decline after 1 year^2^. Using mean scores for whole groups obscures the heterogeneity among stroke survivors. Previous studies modelling cognitive trajectory groups have only lasted up to 1.5 years^6,7^. There is a need for studies of longer follow-up considering that there may be groups of participants following different cognitive trajectories post-stroke.

Emerging experimental evidence suggests that inflammatory mediators post-stroke increase neuronal damage^8^, and it is hypothesized that subacute or chronic global low-grade neuroinflammation may contribute to neurodegeneration in areas remote from the acute lesion^9^. A few studies have also identified systemic inflammation as a possible predictor of cognitive decline^10,11^. Thiel et al. hypothesized that stroke survivors with pre-stroke neurodegeneration would be more vulnerable to cognitive impairment post-stroke, and that those with a prolonged chronic versus a transient acute neuroinflammation post-stroke would decline faster^12^. We have been able to demonstrate in the Nor-COAST study, with analyses of numerous biomarkers in a large ischemic stroke cohort with extended follow-up, that systemic inflammation in the acute phase has the most negative impact on cognitive outcome compared with inflammation measured at 3 and 18 months^13,14^. The link between systemic inflammation and PSCI may hold therapeutic implications. Nonetheless, a comprehensive understanding of whether and how inflammation affects post-stroke cognitive trajectories remains elusive.

In the current study, we aimed to explore whether plasma inflammatory biomarkers and related metabolites were associated with different cognitive trajectories. More specifically, we studied (a) whether plasma inflammatory biomarkers/metabolites in the acute phase and at 3 months post-stroke were associated with particular cognitive trajectory groups up to 36 months post-stroke; (b) whether plasma inflammatory biomarkers/metabolites in the acute phase and after 3 months were associated with changes in cognitive function over time among participants with and without pre-stroke cognitive impairment; and (c) whether premorbid or stroke-related factors confounded these associations.

## Materials and methods

### Study population

The Nor-COAST study is a longitudinal, observational, cohort study, including 815 patients with acute stroke from five Norwegian stroke units from May 2015 through March 2017 and with follow-up assessments at 3, 18 and 36 months^15^. Inclusion criteria were (1) hospitalization within 7 days of symptom debut for acute stroke, (2) stroke diagnosed according to criteria from the World Health Organization or MRI findings of acute cerebral infarction or intracerebral hemorrhage, (3) fluency in a Scandinavian language, (4) being at least 18 years old, and (5) residency within the catchment area of the participating hospitals. The only exclusion criterion was expected survival less than 3 months^15^. Participants in the present study had ischemic strokes and available plasma samples at the acute phase and/or 3 months and had been assessed with the Montreal Cognitive Assessment (MoCA) test at baseline and/or during follow up.

### Clinical evaluation

Information about demographics, comorbidities, the characteristics and treatment of the acute stroke and ongoing infections in the acute phase were collected from medical records. Stroke severity was measured by the National Institutes of Health Stroke Scale (NIHSS) (0–42)^16^. Stroke aetiology and subtype were classified by a modification of Trial of Org 10,172 in Acute Stroke Treatment (TOAST) classification^17^. The modified Rankin Scale (mRS) (0–6) was used for global function^18^. The Charlson comorbidity index (0–37) was calculated to score comorbidity^19^. Finally, a 36-item Frailty Index (FI) (0–1) was calculated to evaluate pre-stroke frailty^20^. Definitions of FI, vascular risk factors (hypertension, atrial fibrillation, hypercholesterolemia, diabetes mellitus) are described in prior publications^13,17,20^. Infections in the acute phase were defined as infections treated with antibiotics during the acute hospital stay.

### Cognitive outcome

MoCA total score (0–30) was used as the primary outcome^21^ with assessments during index stay and 3, 18, and 36 months post-stroke. A difference in MoCA total scores above 2 points was considered clinically relevant^22^. Participants with less than 13 years of education were given one extra point, up to a maximum of 30 points. Participants whom we could not meet in person were assessed with MoCA by telephone^23^. Study nurses classified pre-stroke cognitive status in interviews with caregivers using the global deterioration scale (GDS) (1–7), GDS < 3 were categorised as normal cognition, GDS *≥* 3 were categorised as cognitive impairment^24^.

### Inflammatory biomarkers and metabolites

The dataset of inflammatory biomarkers and related metabolites in the Nor-COAST study have previously been presented in two prior publications^13,14^. We selected a subset of the pro- and anti-inflammatory biomarkers and metabolites related to inflammation, hereafter termed inflammatory biomarkers/metabolites, to use for further analyses in this present study. The selection were based on their association with cognitive outcome^13,14^. The terminal C5b-9 complement complex (TCC), tumour necrosis factor (TNF), interleukin (IL)-1β, IL-1 receptor antagonist (IL-1ra), IL-6, IL-8, macrophage inflammatory protein 1α (MIP-1α), neopterin, quinolinic acid (QA), picolinic acid (Pic), PA ratio = 4-pyridoxic acid / (pyridoxal + pyridoxal 5’-phosphate) (PAr), and HK ratio = (3-hydroxykynurenine / (kynurenic acid + anthranilic acid + xanthurenic acid + 3-hydroxyanthranilic acid) (HKr) (Table 1). Blood samples were obtained during the index hospital stay at median (IQR) Day 4 (3–6) and at 3 months post-stroke (median (IQR) Day 116 (101–140)). Ethylenediaminetetraacetic acid (EDTA) tubes were centrifuged shortly after sampling at 2,000 × *g* for 15 min and frozen immediately at − 80 °C and later stored at − 80 °C in Biobank1^®^, Central Norway Health Authority. The cytokines were analysed by a 27 multiplex cytokine assay and the complement activation product, TCC, by enzyme-linked immunosorbent assay (ELISA), at the Research Laboratory of Nordland Hospital (Bodø, Norway) in 2019^13^. Bevital A/S (Bergen, Norway) analysed neopterin, metabolites of the kynurenine pathway, and B6-vitamers by liquid chromatography–tandem mass spectrometry^14^. Details of handling of the blood samples and methods used for analysis have been described in detail in the Supplementary Information.

### Statistics

Descriptive statistics of the inflammatory biomarkers/metabolites are presented by mean (SD) and median (IQR), and their intercorrelations by Spearman’s rho. Two statistical approaches were used to study associations between inflammatory biomarkers/metabolites and cognitive trajectories (Supplementary Fig. 1). In *the cognitive trajectory group model*, we first investigated whether our stroke population consisted of distinct and meaningful subgroups with similar cognitive trajectories post-stroke based on MoCA total score. We used group-based trajectory modelling (GBTM)^25^, with the Traj plugin for Stata for censored normal distributions^26^. Participants with MoCA in the acute phase and at least one subsequent follow-up were included (*n* = 401). One-way analysis of variance (ANOVA), Kruskal-Wallis test, Pearson’s chi-square test, and Fisher’s exact test were used to test whether characteristics differed between the trajectory groups. The associations between inflammatory biomarkers/metabolites and cognitive trajectory group membership were studied by multinominal logistic regression, with relative risk ratio (RRR) as an effect measure. We also calculated the RRR^IQR^, meaning the RRR when comparing the 25th to the 75th percentile of the biomarker/metabolite. The plasma inflammatory biomarkers/metabolites were analysed one at a time, and the acute phase and 3-month biomarkers/metabolites were analysed separately. The models were adjusted for age, sex, creatinine, and hospital.

In *the cognitive change by pre-stroke cognitive status model*, we studied whether inflammatory biomarkers/metabolites (Table 1) could explain variability in mean change of MoCA total score in the post-stroke period, stratified by normal (GDS < 3) or impaired (GDS ≥ 3) pre-stroke cognitive function. Participants with at least one MoCA assessment were included (*n* = 466). The interaction between inflammatory biomarker/metabolite and time was tested using mixed linear regression. MoCA total scores were used as a dependent variable. The plasma inflammatory biomarkers/metabolites were analysed one at a time, and the acute phase and 3-month biomarkers/metabolites were analysed separately. Fixed effects were biomarkers/metabolites, pre-stroke cognitive function, time as a categorical variable and their two-way and three-way interactions, age, sex, creatinine and hospital. Participants were included as a random effect. A positive coefficient for the interaction term between the inflammatory biomarker/metabolite and time (transitioning from acute phase/3 months to 36 months) was interpreted as higher values of the biomarker/metabolite were associated with improvement or less decline over time. A negative coefficient was interpreted as higher values of the biomarker/metabolite were associated with decline or less improvement over time. Specifically, we showed the results for the 25th and 75th percentiles of the biomarkers/metabolites.

To test the confounding effects of premorbid and stroke-related factors in both models, we separately adjusted for the following covariates: years of education, pre-stroke mRS, Charlson comorbidity index, pre-stroke FI, the modified TOAST classification (five-category covariate), and NIHSS at Day 1. In *the cognitive trajectory group model* with acute-phase biomarkers/metabolites, we further included pre-stroke GDS (continuous) and adjusted for having infections in the acute phase or CRP > 10 mg/L on Day 1 (yes/no) in two additional sets of analyses. In *the cognitive change by pre-stroke cognitive status model* with acute-phase biomarkers/metabolites, we did a set of analyses where we excluded participants with infections in the acute phase or who had C-reactive protein > 10 mg/L on Day 1.

Details regarding statistical models, model testing and criteria for model selection, testing for potential confounders, handling of missing data and extreme values, and statistical assumptions are described in the Supplementary Information. Due to multiple analyses, levels of significance were set to two-tailed p-values < 0.01. All statistical analyses and graphical plots are performed by STATA 18.

### Ethics approval

Participation in the Nor-COAST study was voluntary. Informed consent was obtained from all subjects and/or their legal guardian(s). An oral consent was accepted from patients who were unable to sign a written consent. The study was conducted in accordance with the 1964 Declaration of Helsinki. The main Nor-COAST study as well as the present sub-study were approved by the Regional Committee for Medical and Health Research Ethics in Norway (2015/171/REK Nord; 242042/REK Nord).

## Results

**Fig. 1:**
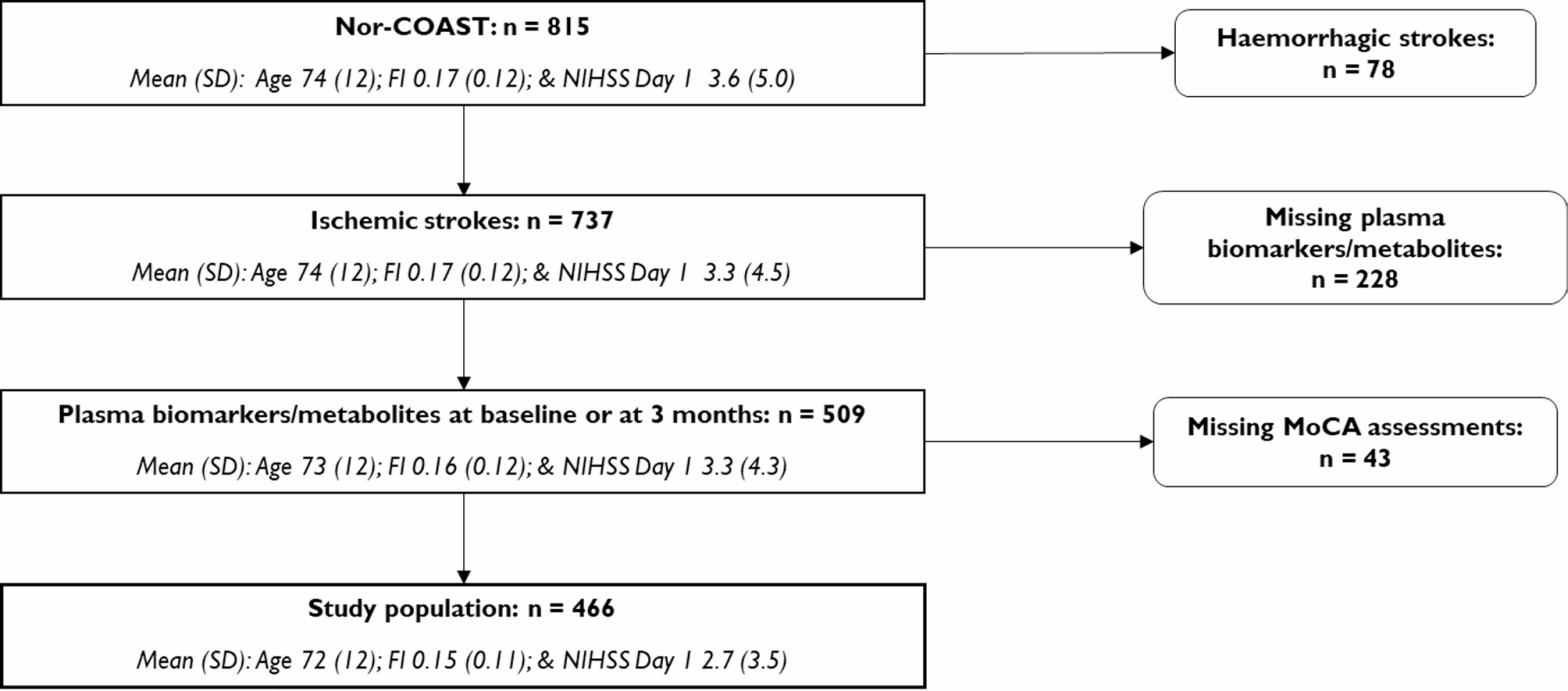
Overview of included participants.

We included 466 participants, 59% males, with mean (SD) age 72 (12) years and 12 (4) years education. Pre-stroke mRS was 0.9 (1.1), pre-stroke FI 0.15 (0.11), and NIHSS at admission and Day 1 4.0 (4.8) and 2.7 (3.5) respectively (Fig. 1; Table 2; Supplementary Fig. 1). Descriptive statistics of inflammatory biomarkers/metabolites and their intercorrelations are given in Supplementary Tables 1 and Supplementary Fig. 2–3.

### The cognitive trajectory group model – the cognitive trajectory groups

**Fig. 2:**
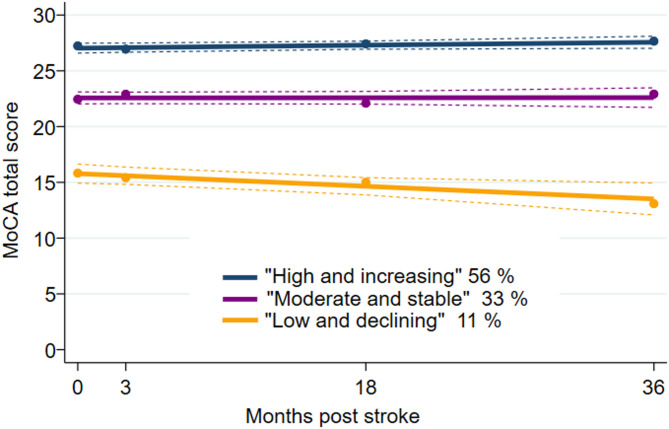
Cognitive trajectory groups of MoCA total scores post-stroke from acute phase to 36 months post-stroke^a^. ^a^Trajectory groups are calculated using group-based trajectory modelling and revealed three groups with shapes 1 1 1 (1 = linear). Group “High and increasing” (navy blue), *n* = 223 (56%), average posterior probability of group membership = 0.95, and odds of correct classification = 14; group “Moderate and stable” (purple), *n* = 133 (33%), average posterior probability 0.89, and odds of correct classification 16; group “Low and declining” (orange), *n* = 45 (11%), average posterior probability 0.94, and odds of correct classification 117.

We found that the best model showed three cognitive trajectory groups with linear development over time (based on MoCA total scores) (Fig. 2). The smallest group was termed “Low and declining” (11%) with a reduction in MOCA total scores over 3 years of 2.2 points, that is considered statistically and clinically significant^22^. The largest group was termed “High and increasing” (56%) and had an increase in MOCA score of 0.72 points over 3 years (statistically, but not clinically significant), and the remaining participants belonged to the “Moderate and stable” group (33%) (Fig. 2; Table 3). The “Low and declining” group consisted of older participants with less education, higher FI and Charlson comorbidity scores, more severe strokes, and with more pre-stroke cognitive impairment (*p* < 0.001) (Table 3). More details on model selection are described in the Supplementary Information (Supplementary Tables 2–3). Supplementary Fig. 4 shows the variability within the groups and illustrates the ceiling effect of MoCA in the “High and increasing” group.

### The cognitive trajectory group model – biomarkers and metabolites in the acute phase

Higher values of the complement marker – TCC, two cytokines – IL-6 and MIP-1α, the marker of cellular immune activation – neopterin, the neurotoxic kynurenine metabolite – QA, and the index based on vitamin B6 metabolites – PAr in the acute phase were associated with increased risk of being in the “Low and declining” group compared to the “High and increasing” group (*p* < 0.01) (Table 4). Higher values of TCC, MIP-1α, neopterin, PAr, and the marker for functional B6-status – HKr were associated with a higher risk of being in the “Moderate and stable” group than in the “High and increasing” group (*p* < 0.01) (Table 4). There were no statistically significant differences between the “Low and declining” group and the “Moderate and stable” group according to levels of biomarkers/metabolites. The pro-inflammatory cytokines TNF, IL-1β, and IL-8, and the anti-inflammatory IL-Ira and the neuroprotective Pic were not associated with group membership.

The RRR^IQR^ for the associations between significant biomarkers/metabolites and the risk of being in the “Low & declining” group compared to the “High and increasing” group, ranged between 1.56 and 3.79 with PAr having the highest RRR^IQR^ (Supplementary Table 4).

Premorbid patient characteristics, and particularly pre-stroke FI, had a greater impact on the analyses than the stroke-related factors and decreased the RRRs and the significance of several of the biomarkers (Supplementary Table 5). The magnitude of the effect of including pre-stroke FI in the model is illustrated by the RRR^IQR^ in Supplementary Table 4. In this model including pre-stroke FI, higher concentrations of TCC remained significantly associated with a higher risk of being in the “Low and declining” group compared to the “High and increasing” group, the significances of IL-6 and PAr were slightly attenuated, but for MIP-1α, neopterin, and QA, the RRRs and significances were considerably attenuated (Supplementary Tables 4–5). Among stroke-related factors, infections in the acute phase and/or elevated CRP at Day 1 had the most impact on the models by attenuating several of the RRRs and their significance, but all p-values remained < 0.05 (Supplementary Table 5).

### The cognitive trajectory group model – biomarkers and metabolites at 3 months

Higher concentrations of neopterin were associated with higher risk of being in the “Low and declining” group compared to the “High and increasing” group, higher values of PAr were associated with a higher risk of being in the “Moderate and stable” group than the “High and increasing” group, while lower concentrations of Pic were associated with a higher risk of being in the “Moderate and stable” group than in the “High and increasing” group (*p* < 0.01) (Supplementary Table 6).

When pre-stroke mRS, pre-stroke GDS, pre-stroke FI, and TOAST categories were separately added as covariates to the models, the RRR and the strength of the association for neopterin at 3 months were attenuated (Supplementary Table 7). Years of education, Charlson comorbidity index, and NIHSS score had less impact on neopterin (Supplemental Table 7).

### The cognitive change by pre-stroke cognitive status model – biomarkers and metabolites in the acute phase

**Fig. 3:**
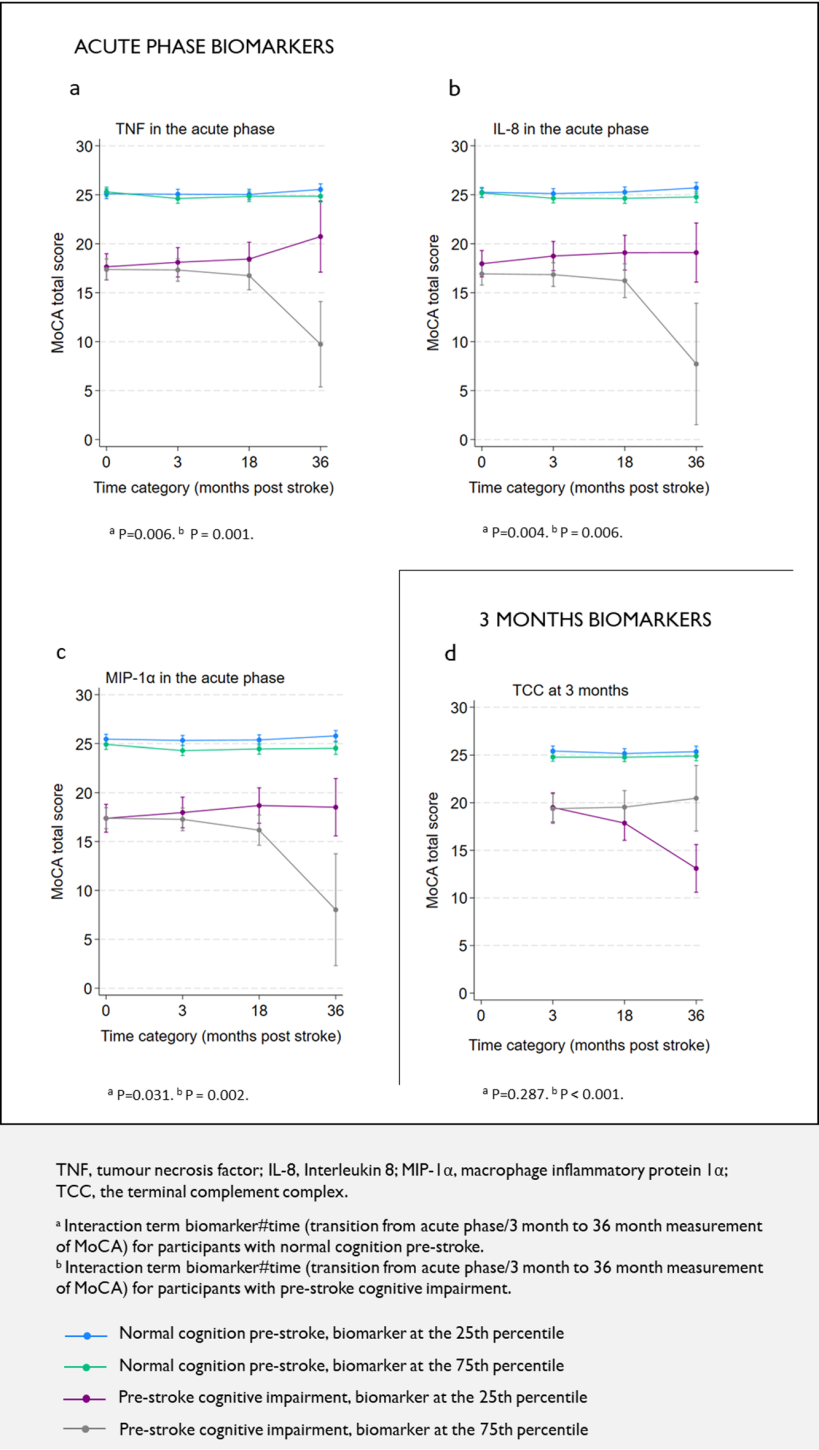
MoCA total scores estimated by mixed linear regression, according the 25th and the 75th percentile of the biomarkers and metabolites, are shown for acute phase (**A**) TNF, (**B**) IL-8, (**C**) MIP-1α, and (**D**) 3 months TCC.

In participants exhibiting normal pre-stroke cognition, higher values of TNF, and IL-8, in the acute phase were associated with a decline or less improvement in MoCA total scores from the acute phase to 36 months (*p* < 0.01) (Fig. 3 Supplementary Fig. 5). Among participants showing pre-stroke cognitive impairment, higher values of TNF, IL-8, and MIP-1α in the acute phase, were associated with a decline or less improvement in MoCA total scores from the acute phase to 36 months (*p* < 0.01) (Fig. 3 Supplementary Fig. 5). Supplementary Table 8 describes numbers and patterns of follow-ups for the participants included in this model.

Separately adjusting for years of education, Charlson comorbidity index and TOAST categories, or excluding participants with infections in the acute phase or CRP > 10 mg/L at Day 1, did not substantially affect the models for participants either with normal or with impaired cognition post-stroke (data only shown for TNF, IL-8 and MIP 1α, Supplementary Fig. 6–8 ). In models separately adjusting for pre-stroke mRS, pre-stroke FI, and NIHSS at Day 1, the slopes in the group of participants with normal cognition pre-stroke remained largely unchanged and the biomarkers remained significant, while among the participants with pre-stroke cognitive impairment, the slopes were only slightly affected, but the significance was attenuated for TNF, IL-8 and MIP-1α (data only shown for TNF, IL-8 and MIP 1α, Supplementary Fig. 6–8).

### The cognitive change by pre-stroke cognitive status model – biomarkers and metabolites at 3 months

Among participants with normal pre-stroke cognition, biomarkers/metabolites were not significantly associated with changes in MoCA total scores over time. Among participants with pre-stroke cognitive impairment, higher values of TCC, IL-1ra, and HKr were associated with improving or a slower decline in MoCA total scores from 3 to 36 months (*p* < 0.01) (Fig. 3 Supplementary Fig. 9). Supplementary Table 9 describes numbers and patterns of follow-ups for the participants included in this model.

These associations remained in analyses adjusted for NIHSS at Day 1, years of education, pre-stroke mRS, Charlson comorbidity index, pre-stroke FI, TOAST category, and NIHSS at Day 1 (data is only shown for TCC, Supplementary Fig. 10).

Pre-stroke cognitive status was significantly associated with a lower MoCA total score in both models including acute and 3 months biomarkers/metabolites (except in the models including Pic and IL-8 at 3 months) (Supplementary Figs. 5 and 9).

## Discussion

In this longitudinal, observational cohort study of patients with acute ischemic strokes over 3 years, we confirmed our hypothesis of associations between systemic inflammation and cognitive trajectories. More specifically, we found that higher values of several biomarkers/metabolites related to acute inflammation were associated with membership in a group characterized by an adverse cognitive trajectory (“Low and declining”). We found that TNF and IL-8 in the acute phase were associated with progressive cognitive decline. The associations between acute-phase biomarkers/metabolites and cognitive trajectory groups were more affected by pre-stroke conditions than by characteristics of the stroke. In particular, pre-stroke frailty seemed to explain parts of the association between acute inflammation and belonging to the “Low and declining” group. Among stroke-related factors, infections in the acute phase were most important, but only explained a smaller part of the association to the “Low and declining” group. While participants with normal pre-stroke cognition had minor change in MoCA total scores over time regardless of inflammation, participants with pre-stroke cognitive impairment had a clinically significant decline in cognition with higher levels of acute systemic inflammation, indicating that this group is especially vulnerable. This declining slope was only slightly affected when adjusting for stroke severity or pre-stroke frailty.

Among biomarkers/metabolites in the acute phase, some (i.e. TCC, IL-6, MIP-1α, neopterin, QA, and PAr) were associated with the “Low and declining” group in *the cognitive trajectory group model*. The group termed “Low and declining” is characterized by low MoCA total score in the acute phase and the associations with inflammatory biomarkers/metabolites may be related to low MoCA total scores in general rather than further decline in cognition over time. This can explain why paradoxically there were other biomarkers (i.e. TNF, IL-8, and MIP-1α,) associated with progressive cognitive decline in *the cognitive change by pre-stroke cognitive status model*. A study by Wang et al. ^10^ found a significant association between higher IL-6 and cognitive decline, not replicated in our study. In their study, blood sampling was performed within 24 h (in contrast to median Day 4 in our population) where the pro-inflammatory effects of IL-6 may be more prominent^27^. In a previous study, we showed that IL-6 increased the risk of PSCI^13^ and in the present study of belonging to the worse cognitive trajectory.

A prior study from Nor-COAST found that FI is strongly associated with the risk of PSCI^20^. Frailty has been claimed to be a predictor of dementia, independently of brain pathology^28^, and frail stroke survivors may be more vulnerable for infections, complications, and other comorbidities during the follow-up that may contribute to cognitive decline. Our results indicate that the associations between IL-6, MIP-1α, neopterin, QA, and PAr and the cognitive trajectory groups could be at least partly explained by pre-stroke frailty. Increased chronic inflammation, and in particular IL-6 but also QA, have been related to frailty^29,30^. Furthermore, a chronic low-grade systemic inflammation associated with ageing and frailty may lead to an altered immune response to stroke in this group, characterized by less efficient phagocytosis and increased secretion of harmful substances^31^. A hypothesis is that inflammation may amplify the adverse effect of frailty, which has been shown in a recent publication that studied the risk of cardiovascular disease^32^. A small study reports higher acute inflammatory responses to trauma among frail individuals, but this study measured inflammatory markers at one single timepoint only, without comparing to basal levels^33^. Consequently, participants in the poorer cognitive trajectory group could be at a slope of cognitive decline even prior to the stroke and responded to the stroke with an increased inflammatory response. Research testing this hypothesis is warranted. Nonetheless, higher concentrations of the complement marker (TCC) in the acute phase remained independently associated with increased risk of belonging to the “Low and declining” group even when adjusting for pre-stroke frailty or pre-stroke cognitive status. TCC is the end product of the complement cascade, and this result indicates that the complement cascade in the acute phase may have detrimental effects and be important in early PSCI. In previous animal studies, especially the upstream complement components (C) 3 and 5 have been associated with the worse outcome related to increased inflammation in the acute phase post stroke^34^. This devastating association is not seen in the subacute and chronic phases where C3 may have a more beneficial role in synaptic plasticity and neurogenesis^34^, which may explain our mixed findings, and even possible protective effect, of TCC at 3 months.

Pre-stroke cognitive status was strongly associated with MoCA total scores at the follow-ups, findings supported by other studies^3,35^. Prior Nor-COAST studies have shown that more pre-stroke brain pathology – such as white matter hyperintensities, medial temporal lobe atrophy, and increased brain-age gap at baseline – is associated with PSCI^36,37^. However, we found that TNF and IL-8 in particular, were associated with progressive decline or less improvement over time in MoCA total scores and that the estimated effect was largest among participants with pre-stroke cognitive impairment. Pre-existing amyloid and tau pathology may be accelerated when the brain is exposed to increased inflammatory stimuli^38^. This group of participants may also be more vulnerable to the inflammatory response as a consequence of aged neurons and already primed microglia^38,39^. Our results should encourage to increased focus on stroke patients with pre-stroke cognitive impairment as these patients may benefit the most from interventions reducing the inflammatory load.

Systemic inflammation appears to have different impact on cognitive trajectories depending on phases post-stroke. We found associations between acute-phase cytokines and cognitive decline. Others have found an association between IL-12 in the subacute phase (median 47 days post-stroke) and cognitive decline up to 5 years after stroke^11^. However, our findings of systemic inflammation at 3 months indicate both positive and negative effects on cognition in this phase. The acute inflammatory reaction is followed by a recovery period including also favourable effects of cytokines and the complement system, consistent with our findings of TCC at 3 months^27,34^. On the other side, atherosclerosis, associated with increased systemic inflammation, may lead to recurrent or persistent ischemia^40^, and may explain why higher concentrations of the anti-inflammatory cytokine IL-1ra at 3 months appear to be protective among participants with pre-stroke cognitive impairment.

The interpretation of our findings is that acute systemic inflammation, representing both premorbid factors, the acute inflammatory response to stroke and stroke-related complications, is important in PSCI. Acute inflammation may lead to increased damage through oxidative stress, release of neurotoxic substances, promoting apoptosis of injured cells, excitotoxicity, and compromised microvasculature^8^. Our results support the hypothesis of Thiel et al. ^12^, which holds that higher levels of inflammation can have a negative impact on cognitive trajectories and that participants with pre-stroke cognitive impairment are most affected. However, we found that systemic inflammation had a more negative impact in the acute phase than at 3 months. Furthermore, these results implies that anti-inflammatory interventions in the acute phase may have the potential to change the slope of cognitive decline. An experimental study showed improved neurological outcome by pre-treating old mice with a TNF-inhibitor before transient cerebral ischemia^41^. Inhibition of complement combined with adhesion molecule inhibition has reduced experimental stroke lesion volume in mice^42^. Complement inhibitors and TNF inhibitors are in routine clinical use for several diseases. Future trials are needed to evaluate the effect of inhibition of TNF and complement in acute stroke.

The major strengths of this study are the prospective design with a large number of participants and with repeated measurements of both inflammatory biomarkers/metabolites and cognitive data. Furthermore, we prepared a solid statistical analysis plan that included two complementary statistical methods for studying trajectories. The advantages of this plan were that we were able to elucidate factors associated with trajectory groups, to study cognitive change, investigate the impact of potential confounders, and show how systemic inflammation in two phases post-stroke influenced cognition. The comprehensive statistical analyses performed contributed to increased understanding of the complex associations between systemic inflammation and cognitive trajectories.

This study comes with some limitations. The participants in Nor-COAST, as well as in this present sub-study, are younger and had milder strokes than the general Norwegian stroke population (Fig. 1)^43^, and therefore, the specific cognitive trajectory groups are not generalizable. In the general Norwegian population, we suspect the group “Low and declining” to be larger than 11%. The cognitive outcome, MoCA total score, is not synonymous to a clinical diagnosis, although Munthe-Kaas et al. showed that MoCA is a valid screening test for post-stroke neurocognitive disorder^44^. The ceiling effect of MoCA may have limited the ability to measure greater improvement over time in the cognitive trajectory group “High and increasing”, as well as limited the difference in MoCA total scores for low and high levels of inflammatory markers among participants with normal cognition pre-stroke. Additionally, the follow-up time may have been too short to find clinically relevant associations between systemic inflammation and change in post-stroke cognition among these participants. More accurate timing of blood sampling and repeated blood sampling in the acute phase would have been preferable as the temporal profile among the cytokines differ and some have pleiotropic effects depending on time after stroke^27,45^. For instance, repeated blood sampling in the acute phase could allowed us to include the important anti-inflammatory cytokine IL-10 that increases in blood 6 days after stroke^45^. The “Low and declining” and “Moderate and stable” groups are small, which may have reduced the statistical power when comparing these two groups. Importantly, the estimated difference in MoCA total scores according to levels of biomarkers/metabolites among participants with pre-stroke cognitive impairment is based on a small group and must be interpreted with caution. Even though we have studied several potential confounders, not all have been addressed. Unfortunately, we did not have sufficient data on recurrent stroke, and a potential confounding effect of recurrent stroke is possible. At last, as we have studied associations, we cannot claim any causal effects. Larger studies are needed to confirm these results, and randomized trials testing anti-inflammatory agents are necessary to prove causality.

## Conclusion

Higher degrees of systemic inflammation in the acute phase of stroke, which may be caused both by premorbid conditions, the response to the acute stroke and stroke-related complications, are associated with worse cognitive trajectories. Decline in cognitive function post-stroke was seen in a group of stroke survivors with low cognitive function in the acute phase post-stroke. Our data suggests that patients with pre-stroke cognitive impairment are particularly vulnerable to the negative effect of acute inflammation on cognitive development post-stroke, and this should be investigated more thoroughly in larger studies. Our results strengthen the basis for future trials on anti-inflammatory treatment in the acute phase of stroke. We encourage researchers to include participants with pre-stroke cognitive impairment and pre-stroke frailty when assessing the effects of systemic inflammation in future studies.

**Table 1:** Overview of inflammatory biomarkers and metabolites included in this study.

Inflammatory biomarkers and metabolites	Description
**The complement system**	
The terminal C5b-9 complement complex (TCC)	End product of the complement cascade ^46^.
**Cytokines**	
Tumour necrosis factor (TNF)	Proinflammatory cytokine ^27^.
Interleukin 1β (IL-1β)	Proinflammatory cytokine ^27^.
Interleukin 1 receptor antagonist (IL-1ra)	Anti-inflammatory cytokine, inhibits IL-1β ^27^.
Interleukin 6 (IL-6)	Pleiotropic cytokine, mainly proinflammatory in the acute phase of stroke but also has anti-inflammatory characteristics ^27^.
Interleukin 8 (IL-8)	Chemokine, attracts mainly neutrophils ^47^.
Macrophage inflammatory protein 1α (MIP-1α)	Chemokine, attracts mainly monocytes^47^.
**Cellular immune activation**	
Neopterin	Neopterin is secreted by macrophages upon stimulation by IFN-γ, produced by Th1 cells, and is considered as a biomarker of cellular immune activation and the IFN-γ signalling pathway^48^.
**The Kynurenine pathway**	
Quinolinic acid (QA)	Neurotoxic kynurenine metabolite contributes to excitotoxicity by activating the *N*-methyl-D-aspartate receptor^49^.
Picolinic (Pic)	Neuroprotective kynurenine metabolite ^49^.
**B6 vitamers and related indexes**	
PA ratio (PAr) *= 4-pyridoxic acid / (pyridoxal + pyridoxal 5*’*-phosphate)*	Biomarker of inflammation. Reflects altered balance of vitamin B6 metabolites during inflammation, which leads to increased PAr^50^.
HK ratio (HKr) = (3-hydroxykynurenine / (kynurenic acid + anthranilic acid + xanthurenic acid + 3-hydroxyanthranilic acid)) *100	Functional vitamin B6 status expressed by a ratio of kynurenine metabolites dependent on the enzymes using pyridoxal 5´-phosphate as a cofactor, inversely related to functional vitamin B6 status, which implies that higher values of HKr are expected during inflammation^51^.

**Table 2:** Baseline characteristics for the included participants.

	*n* = 466		Missing
**Age, mean years (SD)**	72.3	(11.6)	
**Male sex, n (%**)	273	(58.6)	
**Education, mean years (SD**)	12.1	(3.8)	
**Baseline risk factors, comorbidities and function**			
Current cigarette smoking, n (%)	91	(19.6)	2
BMI (kg/m^2^), mean (SD)	26.0	(4.2)	20
Hypertension, n (%)	269	(57.7)	
Hypercholesterolemia, n (%)	223	(47.9)	
Diabetes mellitus, n (%)	97	(20.8)	
Coronary heart disease, n (%)	86	(18.5)	
Atrial fibrillation, n (%)	114	(24.5)	
Previous stroke or TIA, n (%)	108	(23.2)	
Cancer, n (%)	75	(16.1)	
Rheumatic or inflammatory diseases, n (%)	28	(6.0)	
Current depression, n (%)	11	(2.4)	
Renal disease, n (%)	17	(3.7)	
Creatinine in acute phase (µmol/L), mean (SD)	81.3	(32.0)	
Charlson Comorbidity Index, mean (SD)	4.0	(1.9)	
Pre-stroke GDS (1–7), n (%)			4
GDS = 1–2 (normal cognition)	402	(87.0)	
GDS = 3 (Mild Neurocognitive Disorder)	32	(6.9)	
GDS = 4–7 (Major Neurocognitive Disorder)	28	(6.1)	
mRS pre-stroke (0–6), mean (SD)	0.9	(1.1)	3
Frailty Index pre-stroke, mean (SD)	0.15	(0.11)	3
**Assessments during hospital stay**			
NIHSS (0–42) at admittance, mean (SD)	4.0	(4.8)	15
NIHSS (0–42) at day 1, mean (SD)	2.7	(3.5)	9
mRS (0–6) at discharge*, mean (SD)	2.1	(1.2)	2
Barthel Index (0-100) at discharge*, mean (SD)	89.3	(17.5)	1
**TOAST classification, n (%)**			3
Large vessel disease,	104	(23.2)	
Cardioembolic disease	119	(26.6)	
Small vessel disease	113	(25.2)	
Other aetiology	11	(2.5)	
Undetermined aetiology	101	(22.5)	
**Thrombolysis, n (%)**	119	(25.7)	*3*
**Thrombectomy, n (%)**	11	(2.4)	
**Infections treated with antibiotics, n (%)**	49	(10.6)	*5*
**CRP at Day 1 > 10 mg/L, n (%)**	70	(15.0)	

SD, Standard Deviation; BMI, Body Mass Index; TIA, Transient Ischemic Attack; TOAST, Trial of Org 10,172 in Acute Stroke Treatment, modified as described by Aam et al. [17]; GDS: Global Deterioration Scale; NIHSS, National Institutes of Health Stroke Scale; mRS, modified Rankin Scale. *At discharge or day 7 if length of stay extends beyond 7 days.

**Table 3:** Characteristics of the three trajectory groups.

Group characteristics	“Low and declining” (*n* = 45)	“Moderate and stable” (*n* = 133)	“ High and increasing” (*n* = 223)	*p*-value
n (%)	45 (11.2)	133 (33.2)	223 (55.6)	
Intercept	15.8	22.6	27.3	
Slope, MoCA points/month (p-value)	-0.06 (0.012)	0.00 (0.948)	0.02 (0.020)	
Odds of correct classification	117	16	14	
Average posterior probability	0.94	0.89	0.95	
Age, mean years (SD)	81.2 (8.1)	74.8 (10.5)	67.3 (11.1)	< 0.001^a^
Male sex, n (%)	25 (55.6)	79 (59.4)	133 (59.6)	0.876^b^
Education, mean years (SD)	9.9 (3.2)	11.2 (3.4)	13.7 (3.6)	< 0.001^c^
Pre-stroke mRS (0–6), mean (SD)	1.7 (1.3)	0.9 (1.0)	0.6 (0.8)	< 0.001 ^c^
Pre-stroke GDS (1–7), n (%)				< 0.001 ^c^
GDS = 1–2 (normal cognition)	18 (40.0)	120 (91.6)	223 (100)	
GDS = 3 (Mild Neurocognitive Disorder)	16 (35.6)	8 (6.1)	0 (0)	
GDS = 4–7 (Major Neurocognitive Disorder)	11 (24.5)	3 (2.3)	0 (0)	
Charlson comorbidity index (0–37), mean (SD)	5.5 (1.5)	4.2 (1.7)	3.3 (1.8)	< 0.001^a^
Frailty Index, mean (SD)	0.24 (0.12)	0.15 (0.09)	0.11 (0.06)	< 0.001 ^c^
NIHSS (0–42) at day 1, mean (SD)	4.4 (3.5)	2.5 (2.7)	2.1 (3.5)	< 0.001 ^c^
TOAST				0.046 ^b^
Large vessel disease, n (%)	11 (26.2)	30 (23.1)	44 (20.5)	
Cardioembolic stroke, n (%)	17 (40.5)	38 (29.2)	45 (20.9)	
Small vessel disease, n (%)	9 (21.4)	25 (19.2)	68 (31.6)	
Other aetiology, n (%)	0 (0)	4 (3.1)	6 (2.8)	
Undetermined aetiology, n (%)	5 (11.9)	33 (25.4)	52 (24.2)	
Infections in the acute phase, n (%)	7 (15.9)	14 (10.6)	16 (7.2)	0.154^d^

MoCA, Montreal Cognitive Assessment; GDS, Global Deterioration Scale; mRS, modified Rankin scale; NIHSS, National Institutes of Health StrokeScale; TOAST, Trial of Org 10172 in Acute Stroke Treatment, modified as described by Aam et al. [17]. ^a^ One-way analysis of variance (ANOVA). ^b^Pearson chi-square test. ^c^ Kruskal Wallis test. ^d^ Fischer’s exact test

**Table 4:** Multinominal logistic regression models assessing the associations trajectory group membership by plasma inflammatory biomarkers and related metabolites in the acute phase ^a^.

Biomarkers and metabolites in the acute phase	“Low and declining” vs. “High and increasing”			“Moderate and stable” vs. “High and increasing”			“Low and declining” vs. “Moderate and stable”			*n*
	RRR	95% CI	*p*-value	RRR	95% CI	*p*-value	RRR	95% CI	*p*-value	
TCC (CAU)	**27.93**	**4.21** to **185.46**	0.001**	**8.50**	**2.07** to **34.87**	0.003**	3.29	0.63 to 17.20	0.159	*344*
TNF (pg/mL)	1.009	0.995 to 1.023	0.208	1.004	0.995 to 1.014	0.381	1.005	0.992 to 1.018	0.486	*344*
IL-1β (pg/mL)	1.095	0.964 to 1.243	0.163	1.054	0.976 to 1.138	0.178	1.039	0.916 to 1.178	0.553	*344*
IL-1ra (pg/mL)	1.0006	0.9996 to 1.0016	0.235	1.0001	0.9993 to 1.0008	0.853	1.0005	0.9996 to 1.0014	0.267	*344*
IL-6 (pg/mL)	**1.084**	**1.030** to **1.141**	0.002**	1.057	1.013 to 1.103	0.010*	1.026	0.983 to 1.069	0.238	*345*
IL-8 (pg/mL)	1.015	0.984 to 1.047	0.335	1.014	0.994 to 1.035	0.164	1.001	0.972 to 1.032	0.940	*344*
MIP-1α (pg/mL)	**1.255**	**1.059** to **1.487**	0.009**	**1.218**	**1.073** to **1.382**	0.002**	1.030	0.888 to 1.195	0.694	*344*
Neopterin (nmol/L)	**1.100**	**1.028** to **1.177**	0.006**	**1.066**	**1.018** to **1.117**	0.007**	1.032	0.969 to 1.098	0.328	*331*
Quinolinic acid (nmol/L)	**1.0031**	**1.0010** to **1.0051**	0.003**	1.0011	0.9997 to 1.0026	0.124	1.0020	1.0000 to 1.0039	0.047*	*331*
Picolinic acid (nmol/L)	0.991	0.969 to 1.014	0.432	0.993	0.979 to 1.007	0.334	0.998	0.976 to 1.021	0.862	*331*
PAr	**27.87**	**5.82** to **133.57**	< 0.001**	**4.65**	**1.51** to **14.28**	0.007**	5.99	1.43 to 25.07	0.014*	*331*
HKr	1.022	0.998 to 1.047	0.074	**1.023**	**1.006** to **1.039**	0.007**	0.999	0.977 to 1.022	0.957	*331*

RRR, relative risk ratio; CI, confidence interval; TCC, the terminal complement complex; TNF, tumour necrosis factor; IL-1β, Interleukin 1β; IL-1ra, Interleukin 1 receptor antagonist; IL-6, Interleukin 6; IL-8, Interleukin 8; MIP-1α, macrophage inflammatory protein 1α; PAr, PA ratio = 4-pyridoxic acid / (pyridoxal + pyridoxal 5’-phosphate); HKr, HK ratio = (3-hydroxykynurenine / (kynurenic acid + anthranilic acid + xanthurenic acid + 3-hydroxyanthranilic acid)) * 100. * p<0.05. ** p<0.01.^a^ Multinominal logistic regression model with the plasma inflammatory biomarkers and related metabolites in the acute phase as independent variables and group of cognitive trajectories as dependent variable, adjusted for age, sex, creatinine and hospital.

## Supplementary Information

Below is the link to the electronic supplementary material.

Supplementary Material 1

## Data Availability

This study is based on sensitive data which cannot be made publicly available due to Norwegian legal restrictions. A proportion of the data can however be made available from the corresponding author on a reasonable request.

## Abbreviations

APP: Average posterior probability
BIC: Bayesian information criterion
CAU: Complement activation arbitrary units
CRP: C–reactive protein
EDTA: Ethylenediaminetetraacetic acid
ELISA: Enzyme–linked immunosorbent assay
FI: Frailty index
GBTM: Group–based trajectory modelling
GDS: Global deterioration scale
HKr: HK ratio = (3–hydroxykynurenine / (kynurenic acid + anthranilic acid + xanthurenic acid + 3–hydroxyanthranilic acid)
IL: Interleukin
IL-1ra: - Interleukin 1 receptor antagonist
MIP-1α: Macrophage inflammatory protein 1α
NIHSS: National Institutes of Health Stroke Scale
Nor-COAST: Norwegian cognitive impairment after stroke
MoCA: Montreal cognitive assessment
mRS: The modified Rankin scale
OCC: Odds of correct classification
PAr: PA ratio = 4–pyridoxic acid / (pyridoxal + pyridoxal 5’–phosphate)
Pic: Picolinic acid
PSCI: Post–stroke cognitive impairment
TCC: Terminal C5b–9 complement complex
TNF: Tumour necrosis factor
TOAST: Trial of Org 10172 in acute stroke
