# Supplementary material for: Associations between systemic inflammation and cognitive trajectories post-stroke
Source: Sci Rep. 2025 Nov 28;15:42791. doi: 10.1038/s41598-025-27119-1 (PMC12663362; doi:10.1038/s41598-025-27119-1)
Supplement: Supplementary file 1 — Supplementary Material 1 [file 41598_2025_27119_MOESM1_ESM.pdf]

Supplementary Information

Associations between systemic inflammation and cognitive trajectories post-stroke

Heidi Vihovde Sandvig [a,b], Ingvild Saltvedt [b,c], Trine Holt Edwin [d], Stina Aam [c,b], Katinka Nordheim Alme [e], Rannveig Sakshaug Eldholm [b,c], Stian Lydersen [f], Tom Eirik Mollnes [g,h,i], Ragnhild Munthe-Kaas [j,k], Per Magne Ueland [l], Arve Ulvik [l], Torgeir Wethal [m], Anne-Brita Knapskog [d].

[a] Department of Medicine, Kristiansund Hospital, Møre og Romsdal Hospital Trust, Kristiansund, Norway

[b] Department of Neuromedicine and Movement Science, Faculty of Medicine and Health Science, NTNU – Norwegian University of Science and Technology, Trondheim, Norway

[c] Department of Geriatric Medicine, Clinic of Medicine, St. Olavs Hospital, Trondheim University Hospital, Trondheim, Norway

[d] Department of Geriatric Medicine, Oslo University Hospital, Ullevaal, Oslo, Norway

[e] Department of Internal Medicine, Haraldsplass Deaconess Hospital, Bergen, Norway

[f] Department of Mental Health, Faculty of Medicine and Health Science, NTNU – Norwegian University of Science and Technology, Trondheim, Norway

[g] Department of Immunology, Oslo University Hospital and University of Oslo, Oslo, Norway

[h] Research Laboratory, Nordland Hospital, Bodø, Norway

[i] Centre of Molecular Inflammation Research, NTNU – Norwegian University of Science and Technology, Trondheim, Norway

[j] Department of Medicine, Kongsberg Hospital, Vestre Viken Hospital Trust, Drammen, Norway

[k] Department of Medicine, Bærum Hospital, Vestre Viken Hospital Trust, Drammen, Norway

[l] Bevital A/S, Laboratoriebygget, 5021 Bergen, Norway

[m] Department of Stroke, Clinic of Medicine, St. Olavs Hospital, Trondheim University Hospital, Trondheim, Norway

Corresponding author: Heidi Vihovde Sandvig, Heidi.V.Sandvig@ntnu.no

Table of Contents

Methods 4

Inflammatory biomarkers and metabolites 4

Statistics 4

The cognitive trajectory group model 4

The cognitive change by pre-stroke cognitive status model 5

Testing for potential confounders 5

Handling of missing data 6

Handling of extreme values 7

Statistical assumptions 7

Results 8

Study population 8

Supplementary Fig. 1: 8

Description of inflammatory biomarkers and related metabolites 9

Supplementary Table 1: 9

Supplementary Fig. 2 10

Supplementary Fig. 3: 11

Group based trajectory modelling – model selection 12

Supplementary Table 2: 12

Supplementary Table 3: 13

Supplementary Fig. 4: 14

The cognitive trajectory model 15

Supplementary Table 4: 15

Supplementary Table 5: 16

Supplementary Table 6: 17

Supplementary Table 7: 18

The cognitive change by pre-stroke cognitive status model 19

Supplementary Fig. 5: 19

Supplementary Table 8: 21

Supplementary Fig. 6: 22

Supplementary Fig. 7: 24
Supplementary Fig. 8: 26
Supplementary Fig. 9: 28
Supplementary Table 9: 30
Supplementary Fig. 10: 31

Methods

Inflammatory biomarkers and metabolites

Biobanking at baseline were done at all five hospitals, while biobanking at 3- and 18-months follow-up were done at St. Olav Hospital, Bærum Hospital and Ålesund Hospital, due to logistical problems at two remaining hospitals. Blood sampling was collected by venous puncture performed by trained personnel. Ethylenediaminetetraacetic acid (EDTA) tubes were inverted four to five times and centrifuged shortly after sampling (within maximum 6 hours) at 2,000 × *g* for 15 minutes. EDTA plasma was distributed in aliquots of 0,5-1 ml EDTA and directly thereafter (within a maximum of 60 minutes) stored at the inclusion hospital, before later being transferred to Biobank1®, Central Norway Health Authority for storage. Storage temperature was −80 °C. The samples were transported on dry ice in all transports by an approved shipping company, according to current guidelines for transport of human biological material. The samples were thawed only once. The cytokines and complement activation product were analysed all in one batch at the Research Laboratory of Nordland Hospital (Bodø, Norway) in 2019. We used a multiplex cytokine assay (Bio-Plex Human Cytokine 27-Plex Panel; Bio-Rad Laboratories Inc., Hercules, CA) to analyse cytokines, according to the manufacturer’s instructions. The terminal C5b-9 complement complex (TCC) was chosen to gauge complement activation and was analysed using enzyme-linked immunosorbent assay (ELISA), based on a monoclonal antibody as described in detail previously [1]. Neopterin, metabolites of the kynurenine pathway, and B6-vitamers analysed by Bevital AS by liquid chromatography–tandem mass spectrometry [2]. These metabolites were analysed in two batches: plasma from 3 months post-stroke in 2019, and plasma from the acute phase in 2022

Statistics

The cognitive trajectory group model

Group-based trajectory modelling (GBTM) – criteria for model selection

Group-based trajectory modelling allows for more complex patterns of development including both linear and nonlinear slopes over time. Models with linear, quadratic and

cubic function of time were tested. Model criteria were average posterior probability (APP) of group membership at least 0.7, odds of correct classification (OCC) above 5 and group size of at least 10%. Confidence intervals were checked for no overlap to indicate good fit. A Bayesian information criterion (BIC) was used to estimate the goodness of fit and for model selection [3]. A slope was considered significantly different from zero if two-tailed p-values <0.05.

Multinominal logistic regression

Covariates in the model:

Biomarker/metabolite (continuous) – age (continuous) – sex – creatinine (continuous) – hospital (categorical).

The cognitive change by pre-stroke cognitive status model

Mixed linear regression

Covariates in the model:

Fixed effects: *Biomarker/metabolite (continuous) – GDS dichotomous – time (categorical) – biomarker/metabolite*time – GDS dichotomous*time – biomarker/metabolite*GDS dichotomous – biomarker/metabolite*time*GDS dichotomous – age (continuous) – sex – creatinine (continuous) – hospital (categorical)*.

Random effects: *Participant*

Testing for potential confounders

Potential confounders included in additional models, were chosen a priori based on the literature [4-14]. Data are shown where results indicate that there are confounding effects but not shown for suppressor effects. In the cognitive trajectory model, results after adjustment for additional factors are only shown for the comparison for “Low and declining” versus “High and increasing”.

Handling of missing data

Cognitive data

If a participant was missing less than one third of the items of Montreal Cognitive Assessment (MoCA), we imputed a mean score to generate total score based on the participant's points in the tested items. The number of imputed MoCA scores were 9 (2 %) in the acute phase (vision problems n=2, unknown reason n=7) , 14 (3 %) at 3 months (telephone n=11, unknown reason n=3) , 15 (4 %) at 18 months (telephone n = 11, unknown reason n= 4) and 20 (9 %) at 36 months (telephone n = 15, hand motor skills n=3 aphasia n= 1, unknown reason n=1).

Inflammatory biomarkers and metabolites

Our inflammatory biomarkers and metabolites are based on two datasets, and therefore there are discrepancies between methods used for imputation. For cytokines, which were analysed at Research Laboratory Nordland Hospital (Bodø, Norway), a few values were below the lower limit of detection, and a random number from a uniform distribution was imputed as described in Sandvig et al. 2023 [15]. Among metabolites analysed by Bevital A/S, a missing value for pyridoxal (PL) in the acute phase, which is used to calculate PA-ratio, was missing due to values below the lower limit of detection, and was given a value equal to the lowest value for PL in the baseline data [16]. One participant had a missing value for 3-hydroxyanthranilic acid at 3 months, which is used to calculate HKr, that was not replaced because it was missing for an unknown reason, leading to one missing value of HKr at 3 months.

Other covariates

In both study populations, four participants in the acute phase and seven at 3 months had missing values of creatinine and were excluded from the adjusted analyses (Supplementary Fig. 1). In additional analyses, participants were excluded if they were missing any of the additional covariates used in the current analysis (Table 2).

Handling of extreme values

The upper 2 % of values for the inflammatory biomarkers and metabolites were excluded from all models to avoid that a few extreme values of inflammatory biomarkers and metabolites disproportionately influenced the regression results.

Statistical assumptions

The correlation between the covariates included in the same analyses were assessed and no correlation exceeded 0.7 (assessed by Pearson's or Spearman's correlation depending on normality assumption). We considered the normality of residuals after mixed linear regression to be acceptable after visual inspection of qq-plots.

Results

Study population

Supplementary Fig. 1: Overview over number of participants included in the analyses.

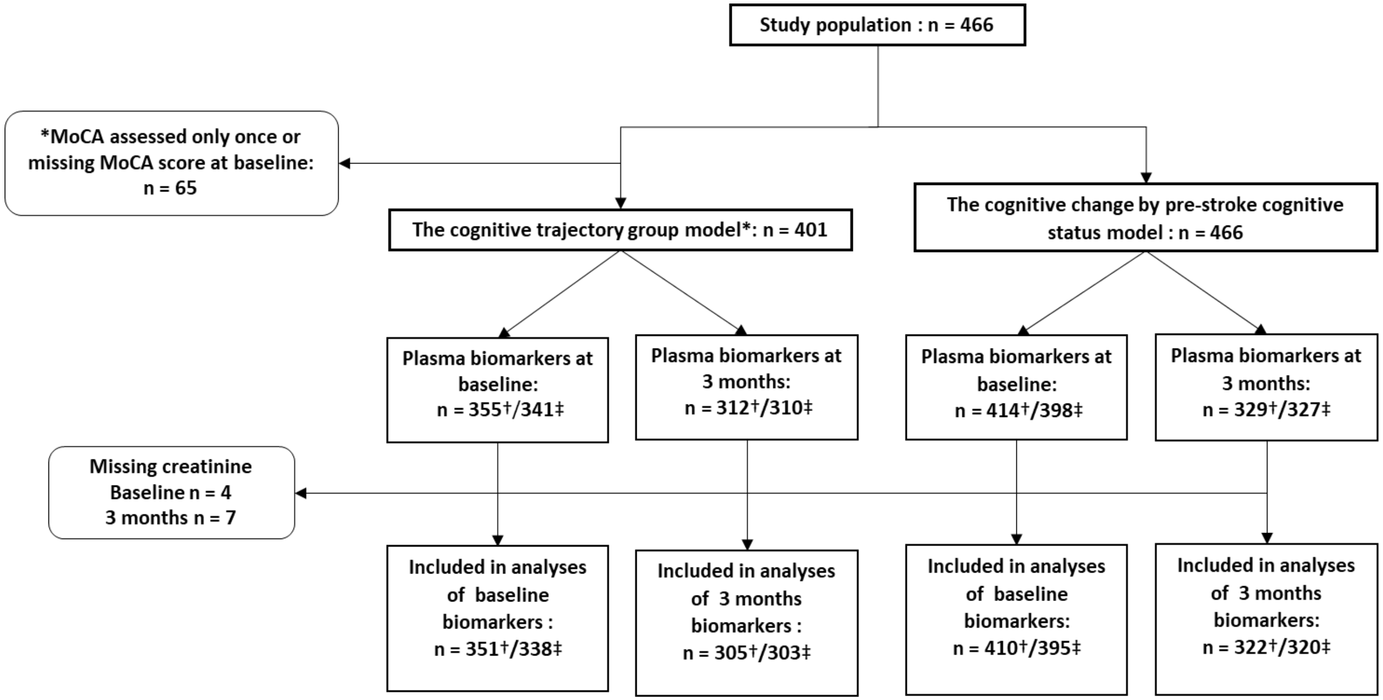

†In analyses of TCC, TNF, IL-1β, IL-6, IL-8, and MIP-1α. ‡In analyses of neopterin, QA, Pic, PAr and HKr.

Description of inflammatory biomarkers and related metabolites

Supplementary Table 1: Descriptive statistics of inflammatory biomarkers and related metabolites in the study population (n=466[a])

	Acute phase				3 months			
	Mean	SD	Median	IQR	Mean	SD	Median	IQR
TCC (CAU)	0.7	0.2	0.7	(0.6 - 0.8)	0.7	0.2	0.7	(0.6 - 0.8)
TNF (pg/mL)	74	31	69	(51 - 90)	73	28	68	(53 - 85)
IL-1β (pg/mL)	3.6	3.3	2.4	(1.5 - 4.8)	2.8	2.2	2.1	(1.3 -3.4)
IL-1ra (pg/mL)	572	460	436	(274 - 740)	419	259	355	(235 - 530)
IL-6 (pg/mL)	8.6	8.2	6.3	(4.0 - 9.8)	5.6	4.1	4.6	(2.9 - 7.1)
IL-8 (pg/mL)	21	15	16	(10 - 29)	20	13	17	(11 - 26)
MIP-1α (pg/mL)	4.8	2.6	4.3	(2.9 - 6.1)	4.2	2.0	3.9	(2.9 - 5.6)
Neopt (nmol/L)	16.3	7.5	14.5	(11.0 - 20.0)	17.4	7.1	15.9	(12.1 - 21.7)
QA (nmol/L)	533	244	244	(365 - 627)	543	217	490	(391 - 648)
Pic (nmol/L)	43	20	39	(28 - 54)	54	21	52	(38 - 66)
PAr	0.6	0.3	0.5	(0.4 - 0.8)	0.7	0.4	0.6	(0.4 - 0.9)
HKr	48	19	44	(36 - 55)	44	16	16	(33 - 50)

TCC, the terminal complement complex; TNF, tumour necrosis factor; IL-1β, Interleukin 1β; IL-1ra, Interleukin 1 receptor antagonist; IL-6, Interleukin 6; IL-8, Interleukin 8; MIP-1α, macrophage inflammatory protein 1α; PAr, PA ratio = 4-pyridoxic acid / (pyridoxal + pyridoxal 5'-phosphate); HKr, HK ratio = (3-hydroxykynurenine / (kynurenic acid + anthranilic acid + xanthurenic acid + 3-hydroxyanthranilic acid)) * 100; RRR, relative risk ratio; CI, confidence interval.
[a] The top 2 % values of each biomarker/metabolite were excluded before the calculations.

Supplementary Fig. 2: Correlation between inflammatory biomarkers and related metabolites in the acute phase by Spearman's rho (n=398)

	TCC (CAU)	TNF (pg/mL)	IL-1β (pg/mL)	IL-1ra (pg/mL)	IL-6 (pg/mL)	IL-8 (pg/mL)	MIP-1α (pg/mL)	Neopt (nmol/L)	QA (nmol/L)	Pic (nmol/L)	PAr	HKr
TCC (CAU)	1.00											
TNF (pg/mL)	0.07	1.00										
IL-1β (pg/mL)	0.12	0.34	1.00									
IL-1ra (pg/mL)	0.16	0.45	0.24	1.00								
IL-6 (pg/mL)	0.23	0.43	0.24	0.43	1.00							
IL-8 (pg/mL)	0.12	0.63	0.48	0.46	0.53	1.00						
MIP-1α (pg/mL)	0.12	0.68	0.39	0.49	0.51	0.72	1.00					
Neopt (nmol/L)	0.14	0.30	0.05	0.22	0.31	0.24	0.33	1.00				
QA (nmol/L)	0.07	0.30	0.07	0.26	0.24	0.17	0.32	0.70	1.00			
Pic (nmol/L)	0.00	-0.10	0.05	-0.11	-0.14	-0.09	-0.14	-0.13	-0.03	1.00		
PAr	0.08	0.24	0.00	0.25	0.31	0.19	0.32	0.53	0.51	-0.11	1.00	
HKr	0.14	0.17	-0.03	0.22	0.37	0.20	0.26	0.39	0.38	-0.33	0.30	1.00

Colour scale: 1 to -1

TCC, the terminal complement complex; TNF, tumour necrosis factor; IL-1β, Interleukin 1β; IL-1ra, Interleukin 1 receptor antagonist; IL-6, Interleukin 6; IL-8, Interleukin 8; Neopt, neopterin; MIP-1α, macrophage inflammatory protein 1α; PAr, PA ratio = 4-pyridoxic acid / (pyridoxal + pyridoxal 5'-phosphate); HKr, HK ratio = (3-hydroxykynurenine / (kynurenic acid + anthranilic acid + xanthurenic acid + 3-hydroxyanthranilic acid)) * 100.

Supplementary Fig. 3: Correlation between inflammatory biomarkers and related metabolites at 3 months by Spearman's rho (n=326)

	TCC (CAU)	TNF (pg/mL)	IL-1β (pg/mL)	IL-1ra (pg/mL)	IL-6 (pg/mL)	IL-8 (pg/mL)	MIP-1α (pg/mL)	Neopt (nmol/L)	QA (nmol/L)	Pic (nmol/L)	PAr	HKr
TCC (CAU)	1.00											
TNF (pg/mL)	0.11	1.00										
IL-1β (pg/mL)	0.09	0.17	1.00									
IL-1ra (pg/mL)	0.19	0.40	0.11	1.00								
IL-6 (pg/mL)	0.22	0.53	0.22	0.45	1.00							
IL-8 (pg/mL)	0.12	0.51	0.41	0.38	0.58	1.00						
MIP-1α (pg/mL)	0.07	0.66	0.36	0.39	0.57	0.68	1.00					
Neopt (nmol/L)	0.15	0.31	0.05	0.10	0.30	0.15	0.31	1.00				
QA (nmol/L)	0.07	0.36	0.13	0.25	0.35	0.23	0.42	0.60	1.00			
Pic (nmol/L)	0.00	0.04	-0.13	-0.06	0.01	0.02	-0.01	0.05	0.04	1.00		
PAr	0.03	0.28	0.07	0.17	0.38	0.24	0.37	0.44	0.45	-0.07	1.00	
HKr	0.02	0.17	-0.01	-0.02	0.22	0.15	0.19	0.42	0.30	-0.20	0.32	1.00

1

-1

TCC, the terminal complement complex; TNF, tumour necrosis factor; IL-1β, Interleukin 1β; IL-1ra, Interleukin 1 receptor antagonist; IL-6, Interleukin 6; IL-8, Interleukin 8; Neopt, neopterin; MIP-1α, macrophage inflammatory protein 1α; PAr, PA ratio = 4-pyridoxic acid / (pyridoxal + pyridoxal 5'-phosphate); HKr, HK ratio = (3-hydroxykynurenine / (kynurenic acid + anthranilic acid + xanthurenic acid + 3-hydroxyanthranilic acid)) * 100.

Group based trajectory modelling – model selection

Models with linear, quadratic and cubic function of time were tested in up to maximum five groups. Bayesian Information Criterion (BIC) was calculated by two different methods based on number of participants (n=401) or number of observations in the model (n=1342). BIC values closer to zero indicate better fit. A five-group model with polynomials 1 3 2 1 2 showed the best fit to our data. When assessing groups size, the smallest group counted less than 10 % of the participants and our criterion of group size was not met (Supplementary Table 2). We then repeated testing with maximum four groups. A four-group model with polynomials 1 1 1 1 showed the best fit, but again the group size criterion was not met (Supplementary Table 2). We then repeated testing with maximum three groups (Supplementary Table 3). A model with polynomials 1 1 1 showed the best fit and met the model criteria. This model had group sizes from 11 % - 56 %, odds of correct classification (OCC) 14.5 - 116.7, and average posterior probability (PP) 0.89 - 0.95 (Supplementary Table 2-3). The three groups were termed "Low and declining" (11%), "Moderate and stable" (33%) and "High and increasing" (56%) according to the mathematical equations of the trajectories.

Supplementary Table 2: Model characteristics for models of cognitive trajectory groups

No of groups	Polynomials	BIC (n= 401)	BIC (n= 1342)	2*ΔBIC (n=401)	Group size (range)	Average PP (range)	OCC
2	1 1	-3552.65	-3556.27		21 % - 79 %	(0.93 - 0.98)	11.1 – 49.4
3	1 1 1	-3452.10	-3457.53	201.1	11 % - 56 %	(0.89 - 0.95)	14.5 – 116.7
4	1 1 1 1	-3427.77	-3435.02	48.66	6 % - 50 %	(0.85 - 0.93)	11.5 – 168.2
5	1 3 2 1 2	-3422.14	-3433.61	11.26	5 % - 47 %	(0.82 - 0.93)	12.2 – 214.1

BIC, Bayesian Information Criterion; PP, posterior probability; OCC, odds of correct classification.

Supplementary Table 3: Comparing the Bayesian Information Criterion (BIC) of models with maximum three groups. The selected model is marked in bold.

Groups	Polynomials	BIC (n= 401)[a]	BIC (n= 1342)[b]
1	1	-3807.19	-3809.00
2	11	-3552.65	-3556.27
**3**	**111**	**-3452.10**	**-3457.53**
3	112	-3455.05	-3461.09
3	113	-3456.71	-3463.36
2	12	-3555.37	-3559.60
3	121	-3453.61	-3459.65
3	122	-3456.58	-3463.22
3	123	-3458.30	-3465.55
2	13	-3558.29	-3563.12
3	131	-3454.58	-3461.23
3	132	-3457.55	-3464.80
3	133	-3458.99	-3466.84
1	2	-3809.59	-3812.01
2	21	-3555.64	-3559.86
3	211	-3454.83	-3460.87
3	212	-3457.78	-3464.42
3	213	-3459.44	-3466.69
2	22	-3558.35	-3563.18
3	221	-3456.28	-3462.92
3	222	-3459.25	-3466.50
3	223	-3460.97	-3468.82
2	23	-3561.27	-3566.71
3	231	-3457.31	-3464.56
3	232	-3460.28	-3468.13
3	233	-3461.72	-3470.18
1	3	-3812.57	-3815.59
2	31	-3558.62	-3563.45
3	311	-3457.63	-3464.28
3	312	-3460.59	-3467.83
3	313	-3462.25	-3470.10
2	32	-3561.33	-3566.77
3	321	-3459.10	-3466.35
3	322	-3462.08	-3469.93
3	323	-3463.79	-3472.25
2	33	-3564.24	-3570.28
3	331	-3460.07	-3467.92
3	332	-3463.04	-3471.49
3	333	-3464.47	-3473.53

Bayesian Information Criterion (BIC) calculated with by number of participants (n=401)[a] or by number of observations (n =1342)[b].

Supplementary Fig. 4: Individual cognitive trajectories in the three trajectory groups.

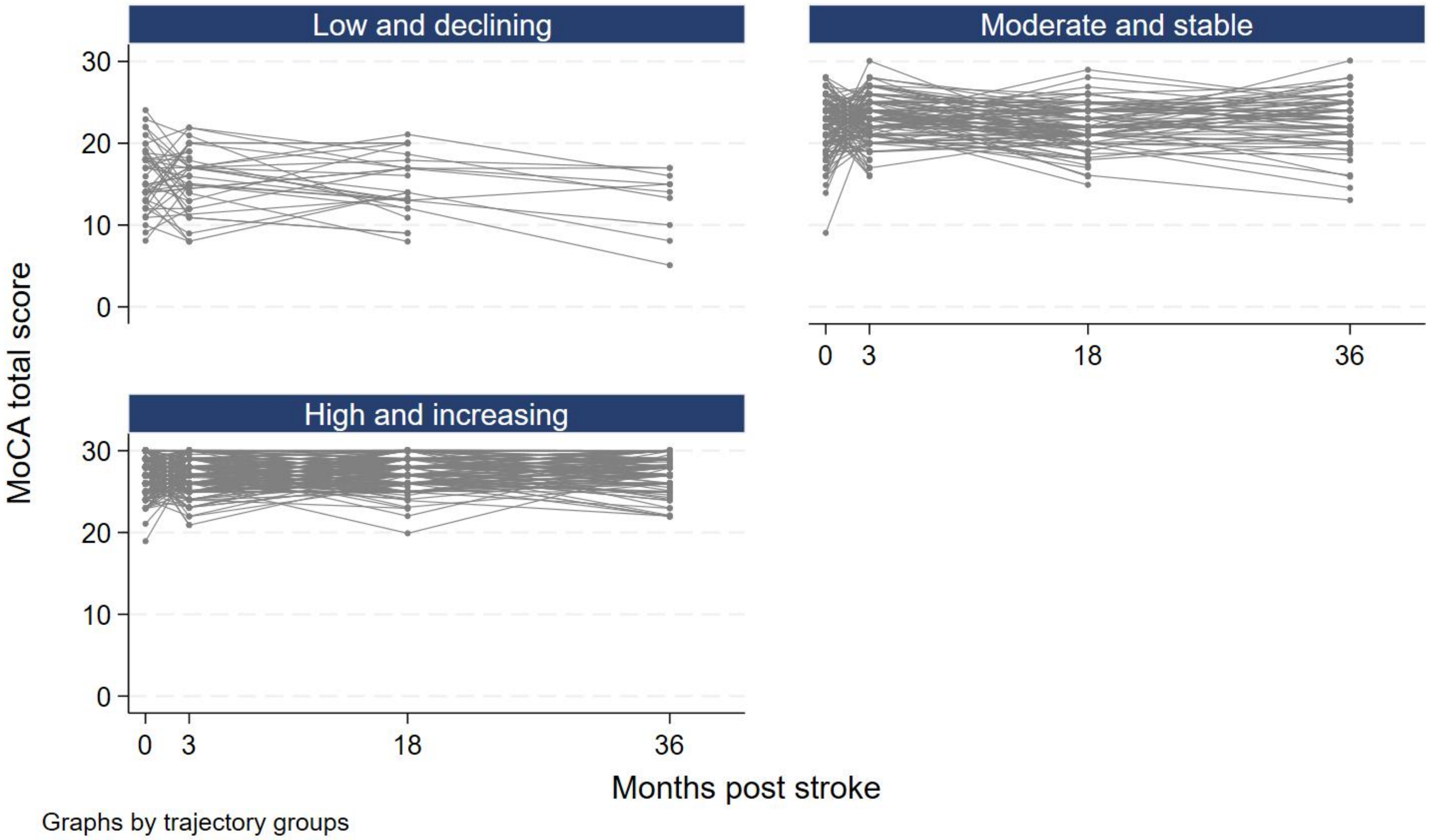

The cognitive trajectory model

Supplementary Table 4: The relative risk ratios (RRR) and the relative risk ratios when increasing from the 25th to the 75th percentile of the biomarkers and metabolites (RRR^{IQR}) for being in group “Low and declining” compared to “High and increasing” by plasma inflammatory biomarkers/metabolites in the acute phase are shown for the main model and the model including Frailty Index as a covariate [a]

		Main		Frailty index	
**Biomarkers and metabolites in the acute phase**	**IQR[a]**	**RRR**	**RRR^{IQR}**	**RRR**	**RRR^{IQR}**
TCC (CAU)	0.2	**27.93****	**1.95****	**17.60****	**1.77****
TNF (pg/mL)	36	1.009	1.37	1.003	1.12
IL-1β (pg/mL)	3.42	1.095	1.36	1.164*	1.68*
IL-1ra (pg/mL)	418	1.0006	1.28	1.0001	1.05
IL-6 (pg/mL)	5.54	**1.084****	**1.56****	1.065*	1.42*
IL-8 (pg/mL)	17.7	1.015	1.31	1.012	1.24
MIP-1α (pg/mL)	2.87	**1.255****	**1.92****	1.125	1.40
Neopterin (nmol/L)	8	**1.100****	**2.14****	1.060	1.60
Quinolinic acid (nmol/L)	259	**1.0031****	**2.22****	1.0020	1.68
Picolinic acid (nmol/L)	26.8	0.991	0.78	1.002	1.05
PAr	0.4	**27.87****	**3.79****	8.831*	2.39*
HKr	18.4	1.022	1.49	0.991	0.84

IQR, interquartile range; RRR, relative risk ratio; TCC, the terminal complement complex; TNF, tumour necrosis factor; IL-1β, Interleukin 1β; IL-1ra, Interleukin 1 receptor antagonist; IL-6, Interleukin 6; IL-8, Interleukin 8; MIP-1α, macrophage inflammatory protein 1α; PAr, PA ratio = 4-pyridoxic acid / (pyridoxal + pyridoxal 5'-phosphate); HKr, HK ratio = (3-hydroxykynurenine / (kynurenic acid + anthranilic acid + xanthurenic acid + 3-hydroxyanthranilic acid)) * 100. [a] IQR is calculated based on the 401 participants included in the cognitive trajectory model and may differ slightly from the data reported in Supplementary Table1. The top 2 % values of each biomarker/metabolite were excluded before the calculations. * p<0.05. ** p<0.01.

[a] Estimates are calculated by multinominal logistic regression adjusted for age, sex, creatinine and hospital (five-category covariate) in the main model, and additional adjustment for Frailty Index in an additional model (right columns).

Supplementary Table 5: Relative risk ratios (RRR) for being in the "Low and declining" group compared to the "High and increasing" group by different biomarkers and metabolites in the acute phase are shown for the main model [a] and for models additionally adjusting for years of education, pre-stroke modified Rankin Scale (mRS), pre-stroke global deterioration scale (GDS), Charlson comorbidity index (CCI), pre-stroke Frailty Index (FI), Trial of Org 10172 in Acute Stroke Treatment (TOAST) classification, National Institutes of Health Stroke Scale (NIHSS) from Day 1, and infections in the acute phase or C-reactive protein (CRP) >10 mg/L on Day 1 (yes/no).

**Biomarkers and metabolites in the acute phase**	***Main***	***Education***	***Pre-stroke mRS***	***Pre-stroke GDS***	***CCI***	***Pre-stroke FI***	***TOAST***	***NIHSS (Day 1)***	***Infections or CRP >10 mg/L***
TCC (CAU)	**27.93****	**24.60****	**25.26****	**173.49****	**18.02****	**17.60****	**41.73****	**25.01****	**19.08****
TNF (pg/mL)	1.009	1.006	1.003	1.008	1.005	1.003	1.013	1.010	1.007
IL-1β (pg/mL)	1.095	1.08	1.129	1.194*	1.132	1.164*	1.113	1.090	1.094
IL-1ra (pg/mL)	1.0006	1.0003	1.0005	1.0015*	1.0005	1.0001	1.0005	1.0004	1.0004
IL-6 (pg/mL)	**1.084****	**1.075****	1.069*	**1.087****	**1.081****	1.065*	**1.085****	**1.083****	1.069*
IL-8 (pg/mL)	1.015	1.014	1.014	1.033	1.014	1.012	1.028	1.017	1.009
MIP-1α (pg/mL)	**1.255****	1.241*	1.148	1.255*	1.214*	1.125	**1.331****	**1.275****	1.228*
Neopterin (nmol/L)	**1.100****	1.085*	1.083*	1.048	1.069	1.060	**1.108****	1.095*	1.086*
Quinolinic acid (nmol/L)	**1.0031****	1.0026*	1.0024*	1.0024	1.0024*	1.0020	**1.0034****	**1.0034****	**1.0030****
Picolinic acid (nmol/L)	0.991	0.991	1.000	0.999	0.994	1.002	0.982	0.993	0.992
PAr	**27.87****	**22.68****	**17.22****	10.18*	**21.81****	8.831*	**25.90****	**33.27****	**22.2****
HKr	1.022	1.017	1.002	1.012	1.016	0.991	1.024	1.026*	1.015

TCC, the terminal complement complex; TNF, tumour necrosis factor; IL-1β, Interleukin 1β; IL-1ra, Interleukin 1 receptor antagonist; IL-6, Interleukin 6; IL-8, Interleukin 8; MIP-1α, macrophage inflammatory protein 1α; PAr, PA ratio = 4-pyridoxic acid / (pyridoxal + pyridoxal 5'-phosphate); HKr, HK ratio = (3-hydroxykynurenine / (kynurenic acid + anthranilic acid + xanthurenic acid + 3-hydroxyanthranilic acid)) * 100. * p<0.05. ** p < 0.01

[a] Adjusted for age, sex, creatinine and hospital.

Supplementary Table 6: Multinominal logistic regression models assessing the associations trajectory group membership by plasma inflammatory biomarkers and related metabolites measured 3 months after stroke [a]

Biomarkers and metabolites at 3 months	"Low and declining" vs "High and increasing"			"Moderate and stable" vs "High and increasing"			"Low and declining" vs "Moderate and stable"			
	RRR	95 % CI	p-value	RRR	95 % CI	p-value	RRR	95 %CI	p-value	*n*
TCC (CAU)	2.095	0.198 to 22.152	0.539	2.703	0.800 to 9.134	0.109	0.775	0.080 to 7.490	0.826	*300*
TNF (pg/mL)	0.995	0.977 to 1.013	0.603	1.001	0.992 to 1.011	0.788	0.994	0.976 to 1.012	0.504	*299*
IL-1β (pg/mL)	0.926	0.733 to 1.171	0.523	0.901	0.786 to 1.033	0.134	1.028	0.811 to 1.303	0.818	*299*
IL-1ra (pg/mL)	1.0000	0.9979 to 1.0021	0.999	1.0010	0.9998 to 1.0022	0.089	0.9990	0.9970 to 1.0010	0.308	*299*
IL-6 (pg/mL)	0.967	0.842 to 1.112	0.641	1.033	0.965 to 1.106	0.348	0.936	0.817 to 1.073	0.344	*299*
IL-8 (pg/mL)	0.982	0.942 to 1.023	0.380	1.004	0.983 to 1.026	0.703	0.978	0.939 to 1.018	0.270	*299*
MIP-1α (pg/mL)	0.951	0.733 to 1.234	0.704	0.975	0.837 to 1.135	0.742	0.976	0.756 to 1.259	0.850	*299*
Neopterin (nmol/L)	**1.127**	**1.043 to 1.218**	**0.002****	1.065	1.011 to 1.123	0.019*	1.058	0.985 to 1.137	0.120	*297*
Quinolinic acid (nmol/L)	1.0014	0.9988 to 1.0039	0.303	1.0011	0.9995 to 1.0027	0.195	1.0003	0.9978 to 1.0028	0.825	*298*
Picolinic acid (nmol/L)	0.988	0.966 to 1.011	0.307	**0.979**	**0.965 to 0.993**	**0.004****	1.010	0.987 to 1.032	0.407	*297*
PAr	1.26	0.27 to 5.83	0.772	**4.27**	**1.69 to 10.76**	**0.002****	0.29	0.07 to 1.27	0.101	*297*
HKr	1.011	0.979 to 1.043	0.512	1.010	0.991 to 1.029	0.312	1.001	0.971 to 1.031	0.959	*297*

RRR, relative risk ratio; CI, confidence interval; TCC, the terminal complement complex; TNF, tumour necrosis factor; IL-1β, Interleukin 1β; IL-1ra, Interleukin 1 receptor antagonist; IL-6, Interleukin 6; IL-8, Interleukin 8; MIP-1α, macrophage inflammatory protein 1α; PAr, PA ratio = 4-pyridoxic acid / (pyridoxal + pyridoxal 5'-phosphate); HKr, HK ratio = (3-hydroxykynurenine / (kynurenic acid + anthranilic acid + xanthurenic acid + 3-hydroxyanthranilic acid)) * 100. * p<0.05. ** p<0.01.

[a] Multinominal logistic regression model with the plasma inflammatory biomarkers and related metabolites at 3 months as independent variables and group of cognitive trajectories as dependent variable, adjusted for age, sex, creatinine and hospital.

Supplementary Table 7: Relative risk ratios (RRR) for being in group "Low and declining" compared to "High and increasing" by different biomarkers and metabolites at 3 months are shown for the main model [a] and for models additionally adjusting for years of education, pre-stroke modified Rankin Scale (mRS), pre-stroke Global Deterioration Scale (GDS), Charlson comorbidity index (CCI), pre-stroke Frailty Index (FI), Trial of Org 10172 in Acute Stroke Treatment (TOAST) classification, and National Institutes of Health Stroke Scale (NIHSS) from Day 1.

**Biomarkers and metabolites at 3 months**	***Main***	***Education***	***Pre-stroke mRS***	***Pre-stroke GDS***	***CCI***	***Pre-stroke FI***	***TOAST***	***NIHSS (Day 1)***
TCC (CAU)	2.095	2.884	1.426	5.505	1.674	1.119	1.829	2.286
TNF (pg/mL)	0.995	0.993	0.999	1.002	0.994	0.997	0.997	0.997
IL-1β (pg/mL)	0.926	0.882	0.840	0.915	0.924	0.875	0.980	0.933
IL-1ra (pg/mL)	1.0000	0.9997	0.9997	1.0002	0.9998	0.999	1.0000	1.0002
IL-6 (pg/mL)	0.967	0.963	0.965	1.049	0.958	0.952	0.978	0.969
IL-8 (pg/mL)	0.982	0.979	0.978	0.998	0.979	0.981	0.990	0.980
MIP-1α (pg/mL)	0.951	0.931	0.948	0.951	0.908	0.903	0.934	0.961
Neopterin (nmol/L)	**1.127****	**1.126****	1.110*	1.107*	**1.116****	1.096*	1.112*	**1.131****
Quinolinic acid (nmol/L)	1.0014	1.0011	1.0016	1.0026	1.0010	1.0014	1.0010	1.0014
Picolinic acid (nmol/L)	0.988	0.991	0.990	0.989	0.990	0.994	0.989	0.982
PAr	1.26	0.95	0.87	0.64	0.79	0.63	0.89	1.48
HKr	1.011	1.005	1.009	0.996	1.003	0.998	1.014	1.011

TCC, the terminal complement complex; TNF, tumour necrosis factor; IL-1β, Interleukin 1β; IL-1ra, Interleukin 1 receptor antagonist; IL-6, Interleukin 6; IL-8, Interleukin 8; MIP-1α, macrophage inflammatory protein 1α; PAr, PA ratio = 4-pyridoxic acid / (pyridoxal + pyridoxal 5'-phosphate); HKr, HK ratio = (3-hydroxykynurenine / (kynurenic acid + anthranilic acid + xanthurenic acid + 3-hydroxyanthranilic acid)) * 100. *p<0.05. **p<0.01.
[a] Adjusted for age, sex, creatinine and hospital.

The cognitive change by pre-stroke cognitive status model

Supplementary Fig. 5: Estimated MoCA according to the 25th and the 75th percentile of the acute phase inflammatory biomarkers and related metabolites, stratified by pre-stroke cognitive status, by mixed linear regression *

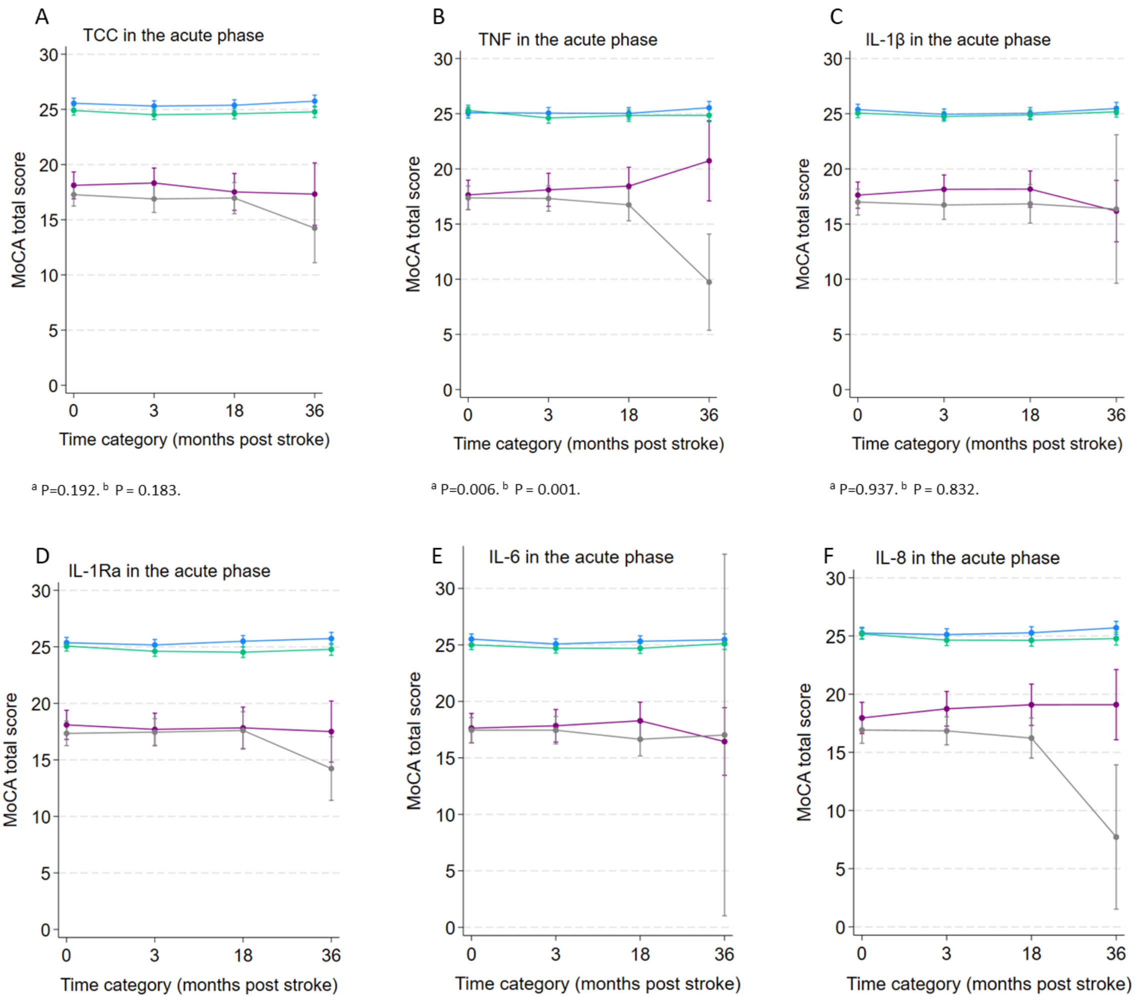

A: [a] P=0.192. [b] P = 0.183.

B: [a] P=0.006. [b] P = 0.001.

C: [a] P=0.937. [b] P = 0.832.

D: [a] P=0.015. [b] P = 0.045.

E: [a] P=0.440. [b] P = 0.918.

F: [a] P=0.004. [b] P = 0.006.

* Mixed linear regression with time as four-category covariate (0, 3, 18 and 36 months), inflammatory biomarker/metabolite in the acute phase, and pre-stroke cognitive status (Global Deterioration Scale [GDS] dichotomous), and their two-way and three-way interactions, hospital, age, sex, and acute phase creatinine level as fixed effects and participant as random effect.

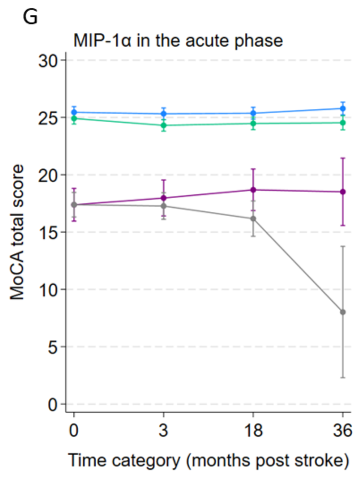

[a] P=0.031. [b] P = 0.002.

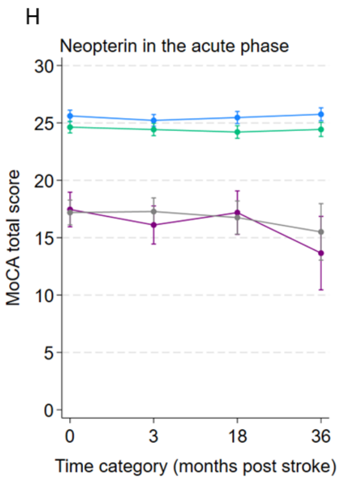

[a] P=0.279. [b] P = 0.029.

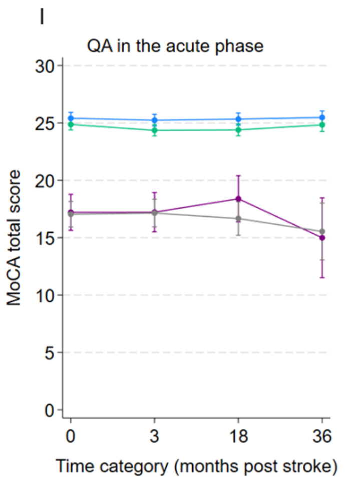

[a] P=0.691. [b] P = 0.570.

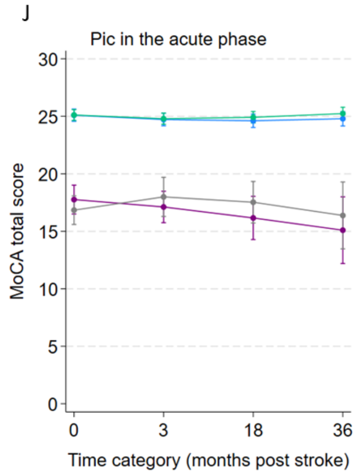

[a] P=0.152. [b] P = 0.146.

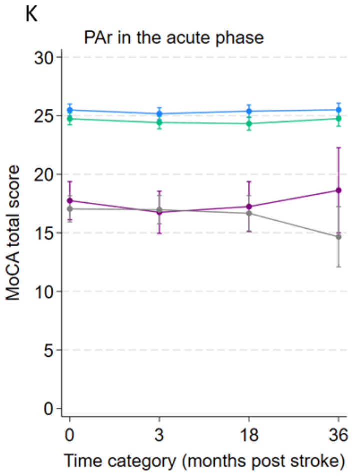

[a] P=0.998. [b] P = 0.050.

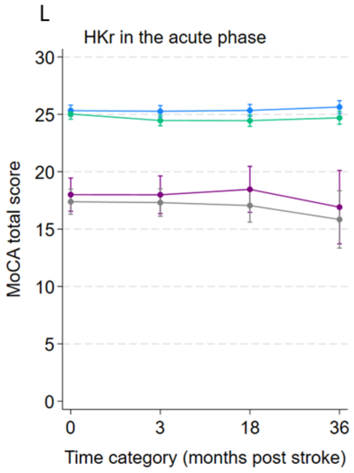

[a] P=0.017. [b] P = 0.577.

TCC, the terminal complement complex; TNF, tumour necrosis factor; IL-1β, Interleukin 1β; IL-1ra, Interleukin 1 receptor antagonist; IL-6, Interleukin 6; IL-8, Interleukin 8; MIP-1α, macrophage inflammatory protein 1α; QA, quinolinic acid; Pic, picolinic acid; PAr, PA ratio = 4-pyridoxic acid / (pyridoxal + pyridoxal 5'-phosphate); HKr, HK ratio = (3-hydroxykynurenine / (kynurenic acid + anthranilic acid + xanthurenic acid + 3-hydroxyanthranilic acid)) * 100.

[a] Interaction term biomarker#time (transition from acute phase to 36 month measurement of MoCA) for participants with normal cognition pre-stroke.

[b] Interaction term biomarker#time (transition from acute phase to 36 month measurement of MoCA) for participants with pre-stroke cognitive impairment.

- Normal cognition pre-stroke, biomarker at the 25th percentile
- Normal cognition pre-stroke, biomarker at the 75th percentile
- Pre-stroke cognitive impairment, biomarker at the 25th percentile
- Pre-stroke cognitive impairment, biomarker at the 75th percentile

Supplementary Table 8: Numbers and patterns of follow-ups among participants included in *the cognitive change by pre-stroke cognitive status model* of acute phase biomarkers/metabolites.

Frequency	Percent	Cumulative percent	Pattern
197	47.58	47.58	1111
79	19.08	66.67	111.
70	16.91	83.57	11..
45	10.87	94.44	1...
6	1.45	95.89	.111
5	1.21	97.10	.1..
4	0.97	98.07	1.11
3	0.72	98.79	1.1.
2	0.48	99.28	.11.
3	0.72	100.00	(other patterns)
**414**	**100.00**		**XXXX**

Supplementary Fig. 6: Estimated MoCA by mixed linear regression*, stratified by pre-stroke cognitive status, according to the 25th and the 75th percentile of acute phase tumour necrosis factor (TNF) adjusted for A) age, sex, creatinine and hospital, and in addition B) years of education, C) pre-stroke modified Rankin scale (mRS), D) Charlson comorbidity index, E) pre-stroke Frailty index, F) modified Trial of Org 10172 in Acute Stroke Treatment (TOAST) classification, G) National Institutes of Health Stroke Scale (NIHSS), and H) excluding participants with infections in the acute phase or who had C-reactive protein >10 mg/L on Day 1.

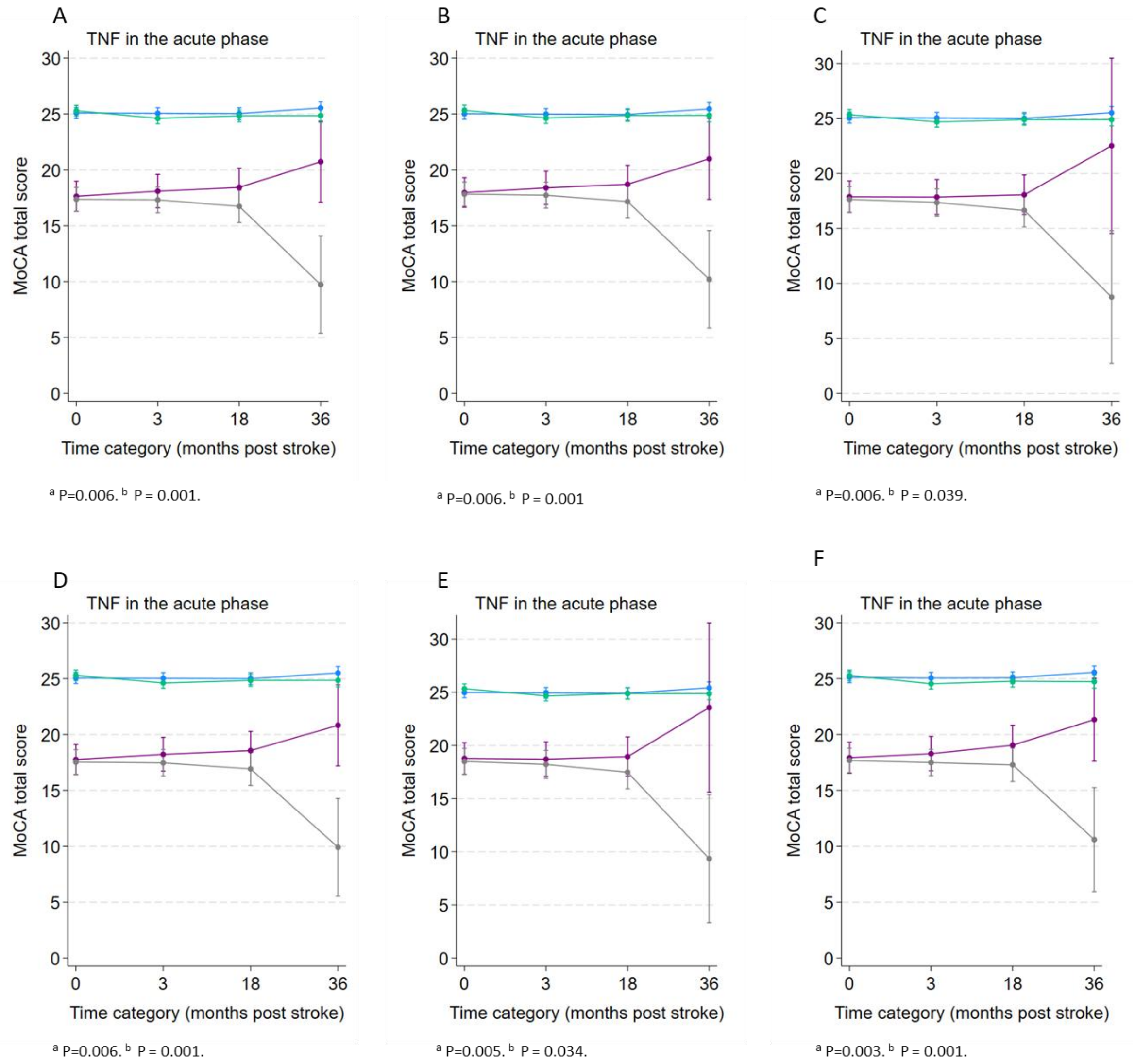

* Mixed linear regression with time as four-category covariate (0, 3, 18 and 36 months), TNF in the acute phase, and pre-stroke cognitive status (Global Deterioration Scale [GDS] dichotomous), and their two-way and three-way interaction as fixed effects, and participant as random effect.

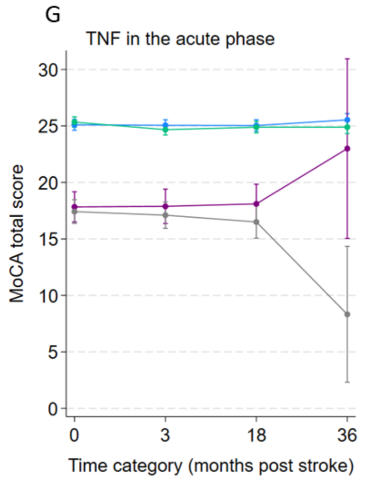

[a] P=0.006. [b] P = 0.030.

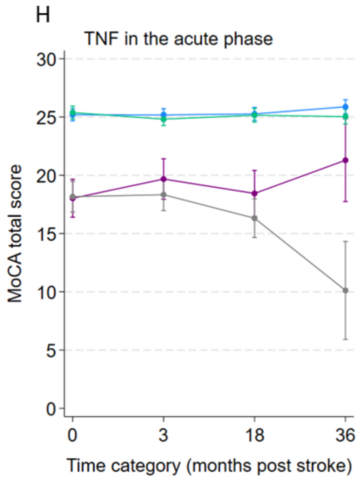

[a] P=0.002. [b] P < 0.001.

TNF, tumour necrosis factor.
[a] Interaction term biomarker#time (transition from acute phase to 36 month measurement of MoCA) for participants with normal cognition pre-stroke.
[b] Interaction term biomarker#time (transition from acute phase to 36 month measurement of MoCA) for participants with pre-stroke cognitive impairment.

- Normal cognition pre-stroke, biomarker at the 25th percentile
- Normal cognition pre-stroke, biomarker at the 75th percentile
- Pre-stroke cognitive impairment, biomarker at the 25th percentile
- Pre-stroke cognitive impairment, biomarker at the 75th percentile

Supplementary Fig. 7: Estimated MoCA by mixed linear regression*, stratified by pre-stroke cognitive status, according to the 25th and the 75th percentile of acute phase interleukin 8 (IL-8) adjusted for A) age, sex, creatinine and hospital, and in addition B) years of education, C) pre-stroke modified Rankin scale (mRS), D) Charlson comorbidity index, E) pre-stroke Frailty index, F) modified Trial of Org 10172 in Acute Stroke Treatment (TOAST) classification, G) National Institutes of Health Stroke Scale (NIHSS), and H) excluding participants with infections in the acute phase or who had C-reactive protein >10 mg/L on Day 1.

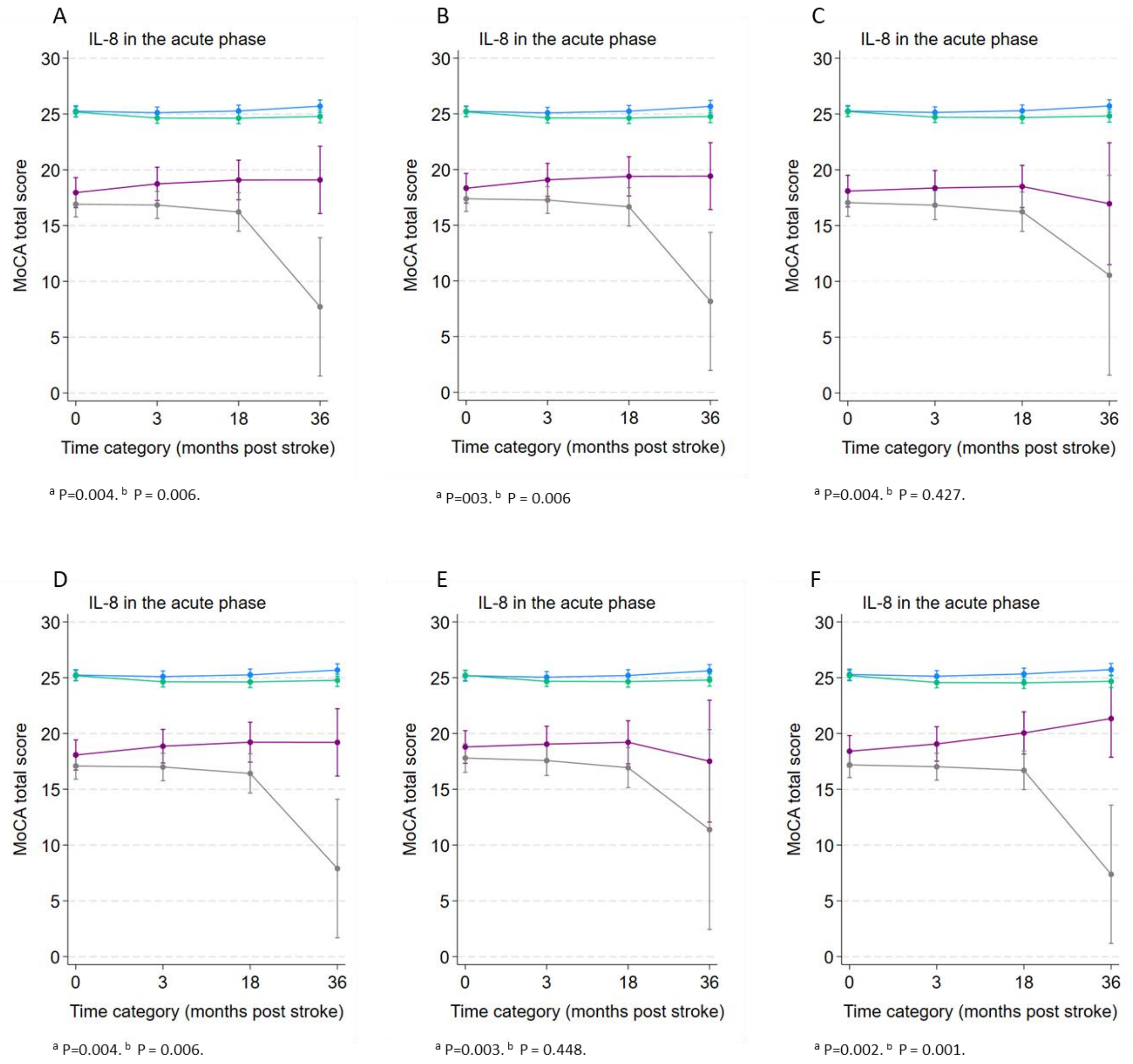

* Mixed linear regression with time as four-category covariate (0, 3, 18 and 36 months), IL-8 in the acute phase, and pre-stroke cognitive status (Global Deterioration Scale [GDS] dichotomous), and their two-way and three-way interactions as fixed effects, and participant as random effect.

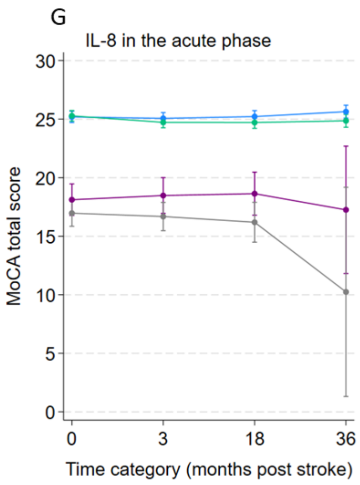

[a] P=0.003. [b] P = 0.386.

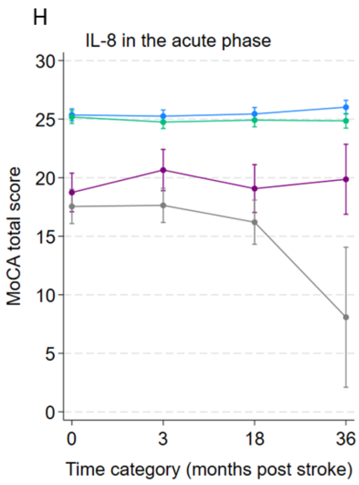

[a] P=0.003. [b] P = 0.004.

IL-8, Interleukin 8
[a] Interaction term biomarker#time (transition from acute phase to 36 month measurement of MoCA) for participants with normal cognition pre-stroke.
[b] Interaction term biomarker#time (transition from acute phase to 36 month measurement of MoCA) for participants with pre-stroke cognitive impairment.

Normal cognition pre-stroke, biomarker at the 25th percentile
Normal cognition pre-stroke, biomarker at the 75th percentile
Pre-stroke cognitive impairment, biomarker at the 25th percentile
Pre-stroke cognitive impairment, biomarker at the 75th percentile

Supplementary Fig. 8: Estimated MoCA by mixed linear regression *, stratified by pre-stroke cognitive status, according to the 25th and the 75th percentile of acute phase macrophage inflammatory protein (MIP-1α) adjusted for A) age, sex, creatinine and hospital, and in addition B) years of education, C) pre-stroke modified Rankin scale (mRS), D) Charlson comorbidity index, E) pre-stroke Frailty index, F) modified Trial of Org 10172 in Acute Stroke Treatment (TOAST) classification, G) National Institutes of Health Stroke Scale (NIHSS), and H) excluding participants with infections in the acute phase or who had C-reactive protein >10 mg/L on Day 1.

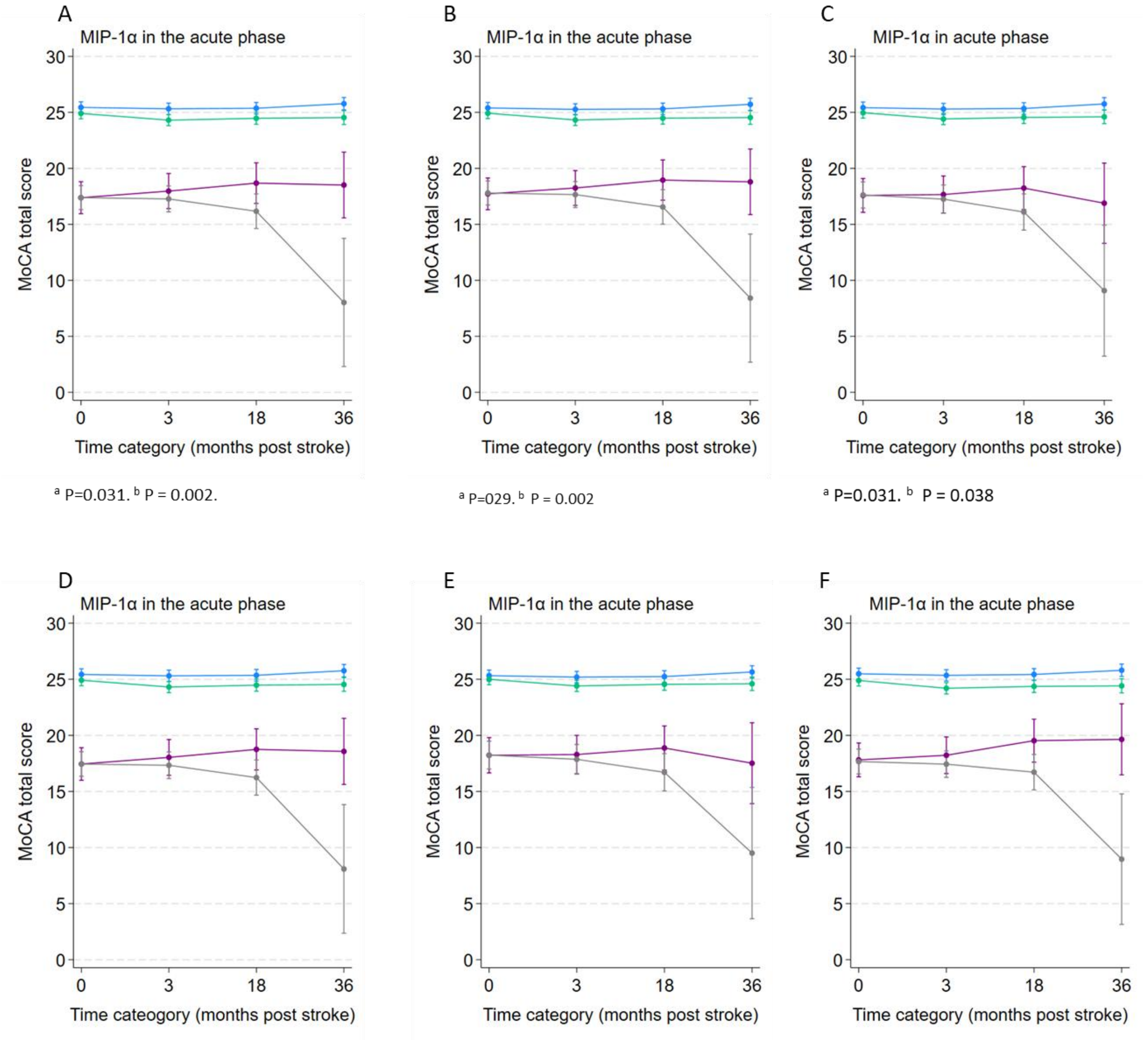

A: [a] P=0.031. [b] P = 0.002.

B: [a] P=029. [b] P = 0.002

C: [a] P=0.031. [b] P = 0.038

D: [a] P=0.031. [b] P = 0.002.

E: [a] P=0.025. [b] P = 0.034.

F: [a] P=0.019 [b] P = 0.002.

* Mixed linear regression with time as four-category covariate (0, 3, 18 and 36 months), MIP-1α in the acute phase, and pre-stroke cognitive status (Global Deterioration Scale [GDS] dichotomous), and their two-way and three-way interactions as fixed effects, and participant as random effect.

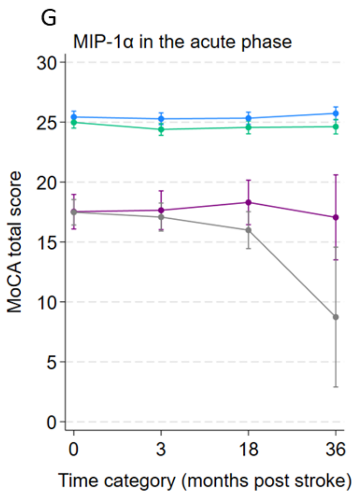

[a] P=0.042. [b] P = 0.029

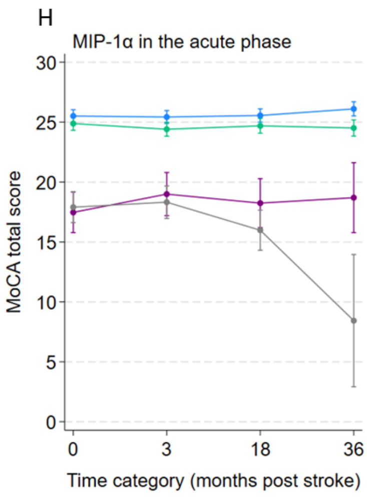

[a] P=0.006. [b] P = 0.001.

MIP-1α, macrophage inflammatory protein
[a] Interaction term biomarker#time (transition from acute phase to 36 month measurement of MoCA) for participants with normal cognition pre-stroke.
[b] Interaction term biomarker#time (transition from acute phase to 36 month measurement of MoCA) for participants with pre-stroke cognitive impairment.

- Normal cognition pre-stroke, biomarker at the 25th percentile
- Normal cognition pre-stroke, biomarker at the 75th percentile
- Pre-stroke cognitive impairment, biomarker at the 25th percentile
- Pre-stroke cognitive impairment, biomarker at the 75th percentile

Supplementary Fig. 9: Estimated MoCA according to the 25th and the 75th percentile of the 3 months inflammatory biomarkers and related metabolites, stratified by pre-stroke cognitive status, by mixed linear regression*

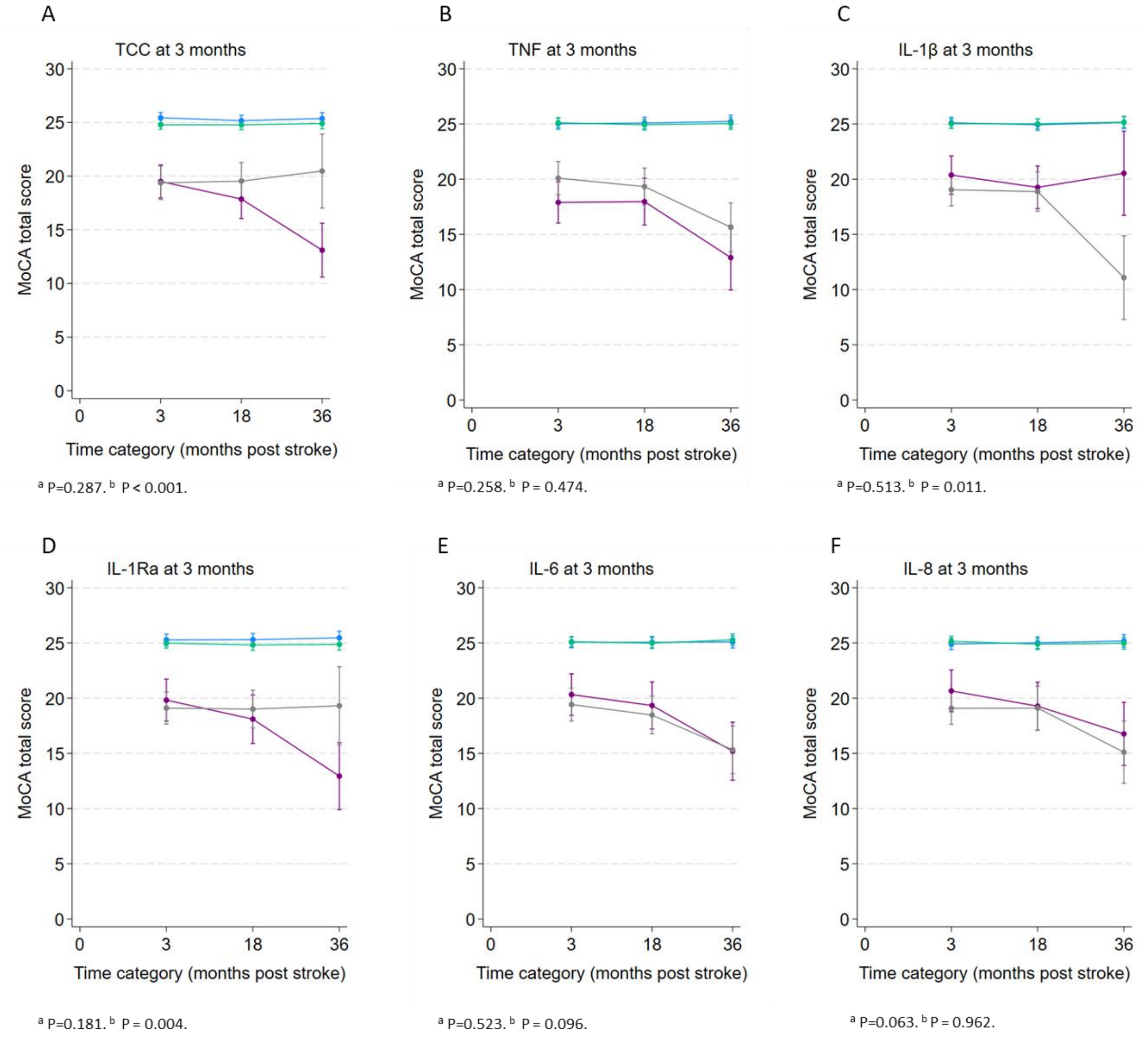

* Mixed linear regression with time as three-category covariate (3, 18 and 36 months), inflammation biomarker/metabolite at 3 months, and pre-stroke cognitive status (Global Deterioration Scale [GDS] dichotomous) and their two-way and three-way interactions, hospital, age, sex, and 3 months creatinine level as fixed effects, and participant as random effect.

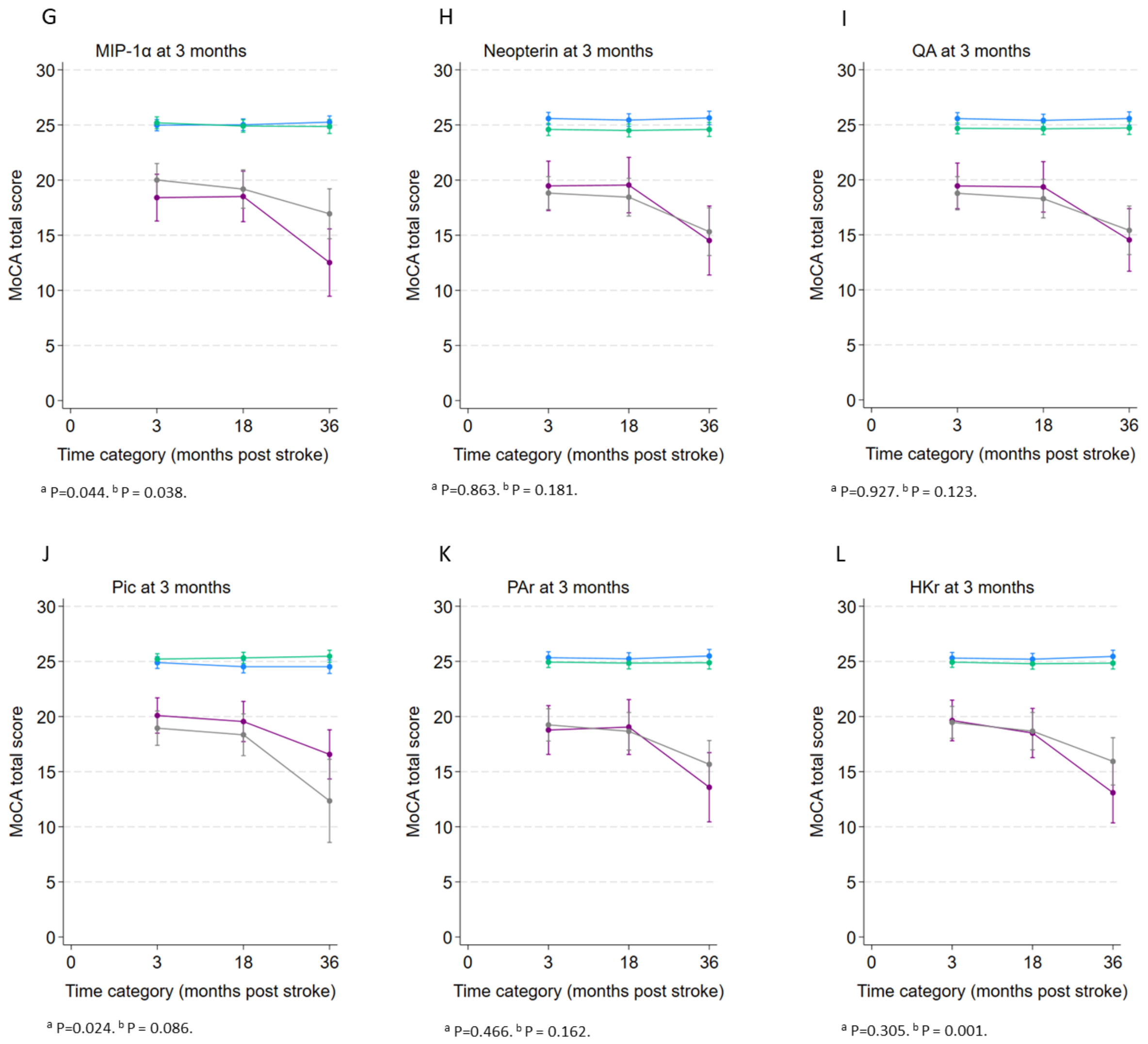

TCC, the terminal complement complex; TNF, tumour necrosis factor; IL-1β, Interleukin 1β; IL-1ra, Interleukin 1 receptor antagonist; IL-6, Interleukin 6; IL-8, Interleukin 8; MIP-1α, macrophage inflammatory protein 1α; QA, quinolinic acid; Pic, picolinic acid; PAr, PA ratio = 4-pyridoxic acid / (pyridoxal + pyridoxal 5'-phosphate); HKr, HK ratio = (3-hydroxykynurenine / (kynurenic acid + anthranilic acid + xanthurenic acid + 3-hydroxyanthranilic acid)) * 100.
[a] Interaction term biomarker#time (transition from first to last measurement of MoCA) for participants with normal cognition pre-stroke.
[b] Interaction term biomarker#time (transition from first to last measurement of MoCA) for participants with pre-stroke cognitive impairment.

- Normal cognition pre-stroke, biomarker at the 25th percentile
- Normal cognition pre-stroke, biomarker at the 75th percentile
- Pre-stroke cognitive impairment, biomarker at the 25th percentile
- Pre-stroke cognitive impairment, biomarker at the 75th percentile

Supplementary Table 9: Numbers and patterns of follow-ups among participants included in *the cognitive change by pre-stroke cognitive status model* of 3 months biomarkers/metabolites.

Frequency	Percent	Cumulative percent	Pattern
190	57.75	57.75	111
79	24.01	81.76	11.
57	17.33	99.09	1..
2	0.61	99.70	1.1
1	0.30	100.00	.1.
**329**	**100.00**		**XXX**

Supplementary Fig. 10: Estimated MoCA by mixed linear regression [*], stratified by pre-stroke cognitive status, according to the 25th and the 75th percentile of 3 months terminal C5b-9 complement complex (TCC), adjusted for A) age, sex, creatinine and hospital, and in addition B) years of education, C) pre-stroke modified Rankin scale (mRS), D) Charlson comorbidity index, E) pre-stroke Frailty index, F) modified Trial of Org 10172 in Acute Stroke Treatment (TOAST) classification, and G) National Institutes of Health Stroke Scale (NIHSS)

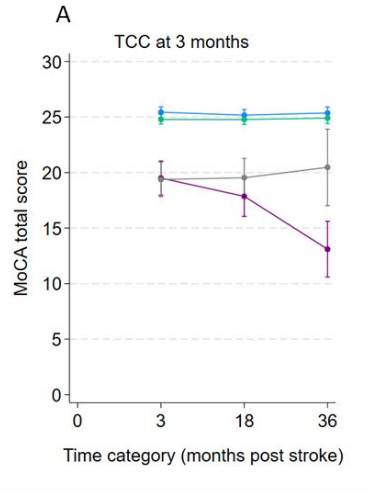

[a] P=0.287. [b] P < 0.001.

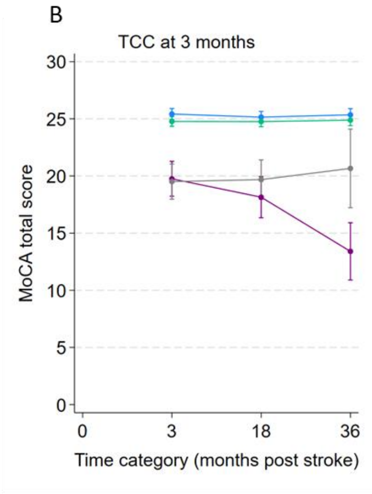

[a] P=0.282. [b] P < 0.001

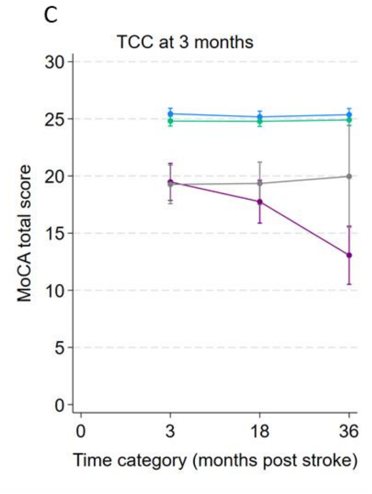

[a] P=0.302. [b] P = 0.004.

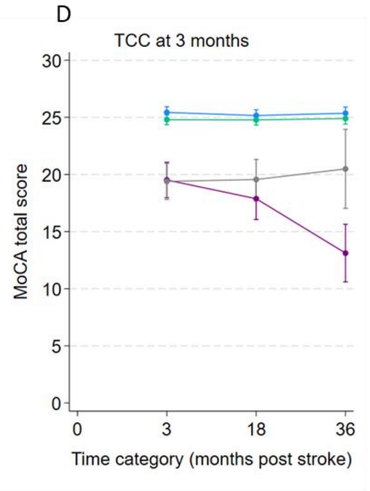

[a] P=0.287. [b] P < 0.001.

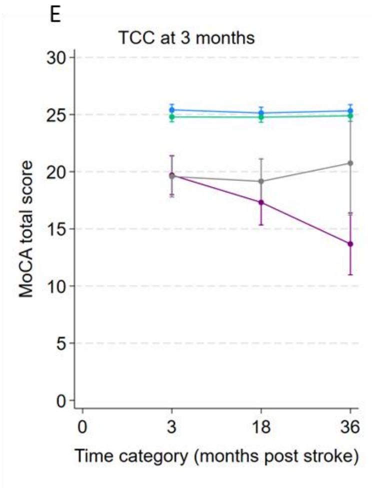

[a] P=0.293. [b] P = 0.003.

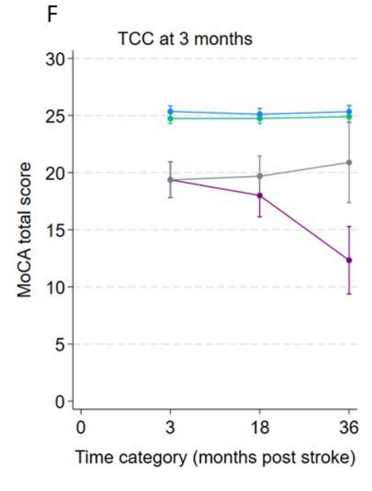

[a] P=0.301. [b] P < 0.001.

[*] Mixed linear regression with time as three-category covariate (3, 18 and 36 months), inflammation biomarker/metabolite at 3 months, and pre-stroke cognitive status (Global Deterioration Scale [GDS] dichotomous) and their two-way and three-way interactions, hospital, age, sex, and 3 months creatinine level as fixed effects, and participant as random effect.

G

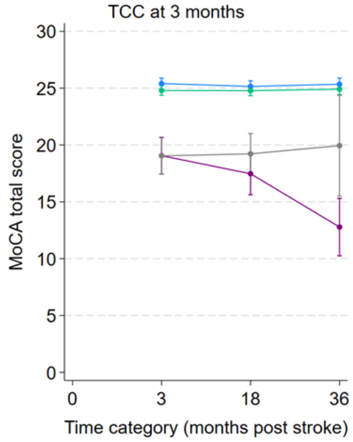

[a] P=0.308. [b] P = 0.004.

TCC, The terminal C5b-9 complement complex
[a] Interaction term biomarker#time (transition from acute phase to 36 month measurement of MoCA) for participants with normal cognition pre-stroke.
[b] Interaction term biomarker#time (transition from acute phase to 36 month measurement of MoCA) for participants with pre-stroke cognitive impairment.

- Normal cognition pre-stroke, biomarker at the 25th percentile
- Normal cognition pre-stroke, biomarker at the 75th percentile
- Pre-stroke cognitive impairment, biomarker at the 25th percentile
- Pre-stroke cognitive impairment, biomarker at the 75th percentile

1 Bergseth, G. *et al.* An international serum standard for application in assays to detect human complement activation products. *Mol. Immunol.* **56**, 232-239 (2013). https://doi.org:10.1016/j.molimm.2013.05.221
2 Midttun, Ø., Hustad, S. & Ueland, P. M. Quantitative profiling of biomarkers related to B-vitamin status, tryptophan metabolism and inflammation in human plasma by liquid chromatography/tandem mass spectrometry. *Rapid Commun. Mass Spectrom.* **23**, 1371-1379 (2009). https://doi.org:10.1002/rcm.4013
3 Nagin, D. S. & Odgers, C. L. Group-Based Trajectory Modeling in Clinical Research. *Annu. Rev. Clin. Psychol.* **6**, 109-138 (2010). https://doi.org:10.1146/annurev.clinpsy.121208.131413
4 Mor, A., Kalaska, B. & Pawlak, D. Kynurenine Pathway in Chronic Kidney Disease: What's Old, What's New, and What's Next? *Int J Tryptophan Res* **13**, 1178646920954882 (2020). https://doi.org:10.1177/1178646920954882
5 Dugué, P. A. *et al.* Association of Markers of Inflammation, the Kynurenine Pathway and B Vitamins with Age and Mortality, and a Signature of Inflammaging. *J. Gerontol. A Biol. Sci. Med. Sci.* **77**, 826-836 (2022). https://doi.org:10.1093/gerona/glab163
6 Solvang, S. H. *et al.* Kynurenine Pathway Metabolites in the Blood and Cerebrospinal Fluid Are Associated with Human Aging. *Oxid. Med. Cell. Longev.* **2022**, 5019752 (2022). https://doi.org:10.1155/2022/5019752
7 Pendlebury, S. T., Rothwell, P. M. & Oxford Vascular, S. Incidence and prevalence of dementia associated with transient ischaemic attack and stroke: analysis of the population-based Oxford Vascular Study. *Lancet Neurol.* **18**, 248-258 (2019). https://doi.org:10.1016/S1474-4422(18)30442-3
8 de Bie, J., Lim, C. K. & Guillemin, G. J. Kynurenines, Gender and Neuroinflammation; Showcase Schizophrenia. *Neurotox. Res.* **30**, 285-294 (2016). https://doi.org:10.1007/s12640-016-9641-5
9 Desmond, D. W., Moroney, J. T., Sano, M. & Stern, Y. Incidence of dementia after ischemic stroke: results of a longitudinal study. *Stroke* **33**, 2254-2260 (2002). https://doi.org:10.1161/01.str.0000028235.91778.95
10 Brouns, R. *et al.* The role of tryptophan catabolism along the kynurenine pathway in acute ischemic stroke. *Neurochem. Res.* **35**, 1315-1322 (2010). https://doi.org:10.1007/s11064-010-0187-2
11 Berger, E. *et al.* Multi-cohort study identifies social determinants of systemic inflammation over the life course. *Nat Commun* **10**, 773 (2019). https://doi.org:10.1038/s41467-019-08732-x
12 Adams, H. P., Jr. *et al.* Baseline NIH Stroke Scale score strongly predicts outcome after stroke: A report of the Trial of Org 10172 in Acute Stroke Treatment (TOAST). *Neurology* **53**, 126-131 (1999). https://doi.org:10.1212/wnl.53.1.126
13 Stanne, T. M. *et al.* Longitudinal Study Reveals Long-Term Proinflammatory Proteomic Signature After Ischemic Stroke Across Subtypes. *Stroke* **53**, 2847-2858 (2022). https://doi.org:10.1161/strokeaha.121.038349
14 Zeng, X. *et al.* Neopterin as a Predictor of Functional Outcome and Mortality in Chinese Patients with Acute Ischemic Stroke. *Mol. Neurobiol.* **53**, 3939-3947 (2016). https://doi.org:10.1007/s12035-015-9310-3

15 Sandvig, H. V. *et al.* Plasma Inflammatory Biomarkers Are Associated With Poststroke Cognitive Impairment: The Nor-COAST Study. *Stroke* (2023). https://doi.org:10.1161/strokeaha.122.041965
16 Sandvig, H. V. *et al.* Neopterin, kynurenine metabolites, and indexes related to vitamin B6 are associated with post-stroke cognitive impairment: The Nor-COAST study. *Brain. Behav. Immun.* (2024). https://doi.org:10.1016/j.bbi.2024.02.030
17 Arrandale, V., Koehoorn, M., MacNab, Y. & Kennedy, S. M. How to use SAS® Proc Traj and SAS® Proc Glimmix in respiratory epidemiology. *Vancouver, BC: University of British Columbia* (2006).
